# Multicomponent Transition Metal Dichalcogenide Nanosheets for Imaging‐Guided Photothermal and Chemodynamic Therapy

**DOI:** 10.1002/advs.202000272

**Published:** 2020-09-30

**Authors:** Yu Zhu, Yingjie Wang, Gareth R. Williams, Liyang Fu, Jingjing Wu, Hui Wang, Ruizheng Liang, Xisheng Weng, Min Wei

**Affiliations:** ^1^ State Key Laboratory of Chemical Resource Engineering Beijing Advanced Innovation Center for Soft Matter Science and Engineering Beijing University of Chemical Technology Beijing 100029 P. R. China; ^2^ Department of Orthopaedics Peking Union Medical College Hospital Peking Union Medical College & Chinese Academy of Medical Sciences Beijing 100730 P. R. China; ^3^ UCL School of Pharmacy University College London 29‐39 Brunswick Square London WC1N 1AX UK

**Keywords:** layered double hydroxide, synergistic therapy, topotactic transformation, transition metal dichalcogenides

## Abstract

Transition metal dichalcogenides (TMDs) have received considerable attention due to their strong absorption in the near‐infrared (NIR) region, strong spin‐orbit coupling, and excellent photothermal conversion efficiency (PCE). Herein, CoFeMn dichalcogenide nanosheets (CFMS NSs) are prepared via facile vulcanization of a lamellar CoFeMn‐layered double hydroxide (LDH) precursor followed by polyvinyl pyrrolidone modification (to give CFMS‐PVP NSs), and found to show excellent photoacoustic (PA) imaging and synergistic photothermal/chemodynamic therapy (PTT/CDT) performance. The as‐prepared CFMS‐PVP NSs inherit the ultrathin morphology of the CoFeMn‐LDH precursor and exhibit an outstanding photothermal performance with a *η* of 89.0%, the highest PCE reported to date for 2D TMD materials. Moreover, 50% of maximum catalytic activity (Michaelis–Menten constant, *K*_m_) is attained by CFMS‐PVP NSs with 0.26 × 10^−3^ m H_2_O_2_ at 318 K, markedly lower than the endogenous concentration of H_2_O_2_ inside tumor cells. In addition, complete apoptosis of HepG2 cancer cells and complete tumor elimination in vivo are observed after treatment with CFMS‐PVP NSs at a low dose, substantiating the NSs’ remarkable PTT/CDT efficacy. This work provides a new and facile approach for the synthesis of high‐quality multicomponent TMD nanosheets with precise process control, the potential for mass production, and outstanding performance, providing great promise in cancer theranostics.

## Graphical abstract

A highly tunable polyvinyl pyrrolidone modified CoFeMn dichalcogenide nanosheets (CFMS‐PVP NSs) platform is fabricated via facile vulcanization of lamellar CoFeMn‐layered double hydroxide precursor, which shows outstanding photothermal conversion efficiency (*η* = 89.0%), effective photoacoustic imaging and synergistic photothermal/chemodynamic therapy.

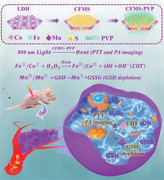

## 1 Introduction

Transition metal dichalcogenides (TMDs) have received considerable attention due to their tunable bandgaps, strong spin‐orbit coupling, and excellent optical and thermal conversion efficiency.^[^ ^1^ ^]^ For example, MoS_2_, WS_2_, and NiTe_2_ show strong absorption in the near‐infrared (NIR) region, which makes them ideal photoacoustic (PA) imaging contrast agents and photothermal therapy (PTT) agents.^[^ ^2^ ^]^ In order to achieve suitable photothermal therapeutic performance, the key factor is to fabricate high‐efficiency photothermal agents. In contrast to the multilayer structure of bulk TMDs, ultrathin TMD nanosheets possess a higher photothermal conversion efficiency (PCE) due to the change of band structure.^[^ ^3^ ^]^ Moreover, compared with mono‐ and dual‐metal TMD NSs, the high entropy induced by multi‐metal coordination endows TMD NSs with tunable and enhanced intrinsic optical and electronic properties, which is of benefit for PTT efficiency.^[^ ^4^ ^]^ Nevertheless, it is still difficult to completely eradicate deep tumors and prevent metastasis by PTT alone, due to the fact that heat dispersion within the solid tumor is inhomogeneous and because of the depth‐dependent decline of laser intensity upon external irradiation.^[^ ^5^ ^]^ Therefore, modulating composition and structure to increase PTT effectiveness, and combining PTT with other therapeutic modalities, are vital to fulfill their potential in cancer theranostics.

Chemodynamic therapy (CDT) utilizes the iron‐initiated Fenton reaction or Fenton‐like reactions mediated by other metal ions (such as Co^2+^, Ti^3+^, Ni^2+^, Cu^2+^, and Mn^2+^) to specifically eliminate tumor cells by converting intracellular H_2_O_2_ into toxic hydroxyl radicals (·OH) in acidic conditions. This surmounts issues of tumor nonspecificity and the toxicity of chemotherapeutic drugs.^[^ ^6^ ^]^ However, the fact that glutathione (GSH), which has ·OH scavenging ability, is overexpressed in the tumor microenvironment and has restricted the application of CDT to some degree.^[^ ^7^ ^]^ Thus, the reduction of intracellular GSH level is highly desirable for potent CDT. Moreover, previous reports indicate that external energy fields such as heat could be explored to accelerate Fenton and Fenton‐like reactions and increase CDT efficacy.^[^ ^8^ ^]^ The combination of PTT and CDT will result in effective synergistic cancer therapy, overcoming the inadequacies of any single therapy.^[^ ^9^ ^]^ Therefore, the preparation of TMDs containing photothermal and chemodynamic dual‐functional metal species should allow construction of an “all in one” platform to realize imaging‐guided PTT/CDT. However, there are few reports on multicomponent ultrathin TMDs for biomaterial applications, partly due to limitations in terms of preparation. In stark contrast to TMDs, layered double hydroxide (LDH) nanosheets can be synthesized with very high degrees of structural and compositional control, and at large scale.^[^ ^10^ ^]^ LDH nanosheets are thus widely explored 2D nanomaterials. Their chemical formula can be expressed as [M^2+^_1−_ *_x_*M^3+^ *_x_*(OH)_2_](A*^n−^*)*_x/n_* ·*m*H_2_O, where M^2+^ and M^3+^ are metal ions distributed in an edge‐sharing MO_6_ octahedral host layer.^[^ ^11^ ^]^ A topotactic transformation reaction can be envisaged which might allow the synthesis of ultrathin TMDs from an LDH precursor.^[^ ^12^ ^]^ The resulting TMD nanosheets would inherit the intrinsic architecture, structure and composition of the LDH precursor. Hence, the TMD's properties could be finely tuned through modulating the LDH precursor and transformation conditions.

Herein, polyvinyl pyrrolidone (PVP) modified CoFeMn dichalcogenide nanosheets (CFMS‐PVP NSs) are prepared via facile vulcanization of a lamellar CoFeMn‐LDH precursor for the first time, and show excellent PA imaging and PTT/CDT synergistic therapy performance. X‐ray diffraction (XRD) patterns show the CFMS NSs to comprise a hybrid crystal structure of FeS_2_ and CoS_2_, while transmission electron microscope (TEM) and atomic force microscope (AFM) images reveal ultrathin structures with an average size of ≈54.0 nm and a thickness of ≈1.2 nm. The PCE (*η*) of the CFMS‐PVP NSs can reach 89.0% through modulation of the Co/Fe/Mn ratio in the LDH precursor, the highest PCE ever reported among 2D TMDs. The CFMS‐PVP NSs could convert H_2_O_2_ in situ into toxic ·OH via Fenton and Fenton‐like reactions of Co^2+^ and Fe^2+^/Fe^3+^, with an ultralow Michaelis–Menten constant (*K*_m_ = 0.26 × 10^−3^ m at 318 K) while the Mn^4+^/Mn^3+^ in the CFMS‐PVP NSs could consume GSH rapidly through a redox reaction. Moreover, the NSs have excellent PA imaging capabilities with an ultralow detection limit, as is demonstrated in vivo. Both in vitro and in vivo tests (including conventional and in situ tumor models) substantiate remarkable PTT/CDT efficacy, with complete apoptosis of HepG2 cancer cells and complete tumor elimination after treatment with CFMS‐PVP NSs.

## 2 Result and Discussion

### 2.1 Synthesis and Characterization

**Scheme 1:**
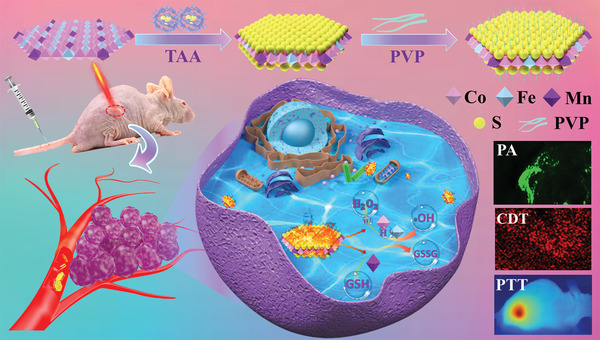
A schematic illustration for preparing CFMS‐PVP NSs to give efficient PTT/CDT and PA imaging.

**Figure 1:**
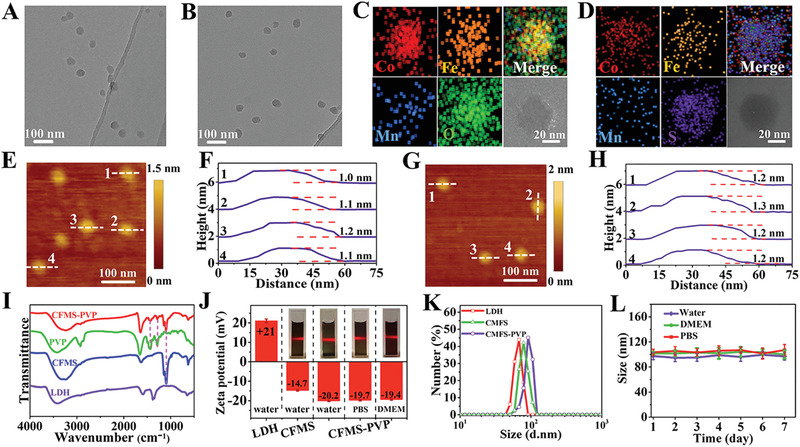
Structure and physicochemical properties of the Co_2_Fe_0.75_Mn_0.25_‐LDH and Co_2_Fe_0.75_Mn_0.25_S_6_ samples. A,B) TEM images, C,D) EDX mapping, E–H) AFM images of Co_2_Fe_0.75_Mn_0.25_‐LDH and Co_2_Fe_0.75_Mn_0.25_S_6_ NSs, respectively. I) FT‐IR spectra. J) Zeta potential and K) size distributions. L) Particle sizes in water, PBS or DMEM for 7 days (*n* = 3, mean ± S.D).

The fabrication of the CFMS‐PVP NSs is illustrated in **Scheme** **1**. Lamellar CoFeMn‐LDH precursors were first prepared by a “bottom‐up” synthesis route, followed by a facile vulcanization process to obtain ultrathin CFMS NSs.^[^ ^13^ ^]^ The CoFeMn‐LDH precursors have high crystallinity, as demonstrated by their XRD patterns (Figure S1, Supporting Information), which show a series of (00l) reflections. After vulcanization, the XRD patterns of the CFMS NSs (Figure S2, Supporting Information) display Bragg reflections which can be ascribed to cubic FeS_2_ (JCPDS Card No. 42‐1340) and CoS_2_ structures (JCPDS Card No. 41‐1471). A TEM image of the Co_2_Fe_0.75_Mn_0.25_‐LDH precursor shows a monodispersed nanosheet morphology, with a lateral size of 50.0 ± 4.5 nm (**Figure** **1**A). After vulcanization, the resulting CFMS NSs inherit the plate‐like morphology and particle size of the precursor, with a diameter 54.0 ± 5.1 nm (Figure 1B). Energy‐dispersive X‐ray (EDX) mapping of the Co_2_Fe_0.75_Mn_0.25_‐LDH precursor reveals a homogeneous distribution of Co, Fe, Mn, and O (Figure 1C), while a uniform dispersion of Co, Fe, Mn, and S is observed for the CFMS NSs (Figure 1D). Thus, it is proposed that CFMS NSs possess a CoS_2_/FeS_2_ crystal phase with Mn doping into the lattice. An AFM image of the Co_2_Fe_0.75_Mn_0.25_‐LDH precursor gives a thickness of ≈1.1 nm (Figure 1E,F). This ultrathin structure is maintained after vulcanization, and the CFMS NSs have an unaltered thickness of ≈1.2 nm (Figure 1G,H).

In order to enhance dispersion stability and biocompatibility for further experimental studies, the surface of the Co_2_Fe_0.75_Mn_0.25_S_6_ NSs was functionalized with PVP. Fourier transform infrared spectroscopy (FT‐IR) was performed to verify the synthesis of LDH precursor, CFMS NSs and CFMS‐PVP NSs (Figure 1I). The LDH spectrum contains a broad band at 3420 cm^−1^, corresponding to the stretching mode of hydroxyl groups. The broad intense band at around 1384 cm^−1^ is attributed to N–O vibrations of nitrate ions. The CFMS spectrum presents no nitrate bands, but instead M–S vibrations are visible in the low wavelength region. C–N stretching bands at 1423 and 1290 cm^−1^ are observed with CFMS‐PVP NSs, which indicates PVP is successfully loaded onto the NSs.

X‐ray photoelectron spectroscopy (XPS) was employed to characterize the chemical composition and valence state of the samples. For the Co_2_Fe_0.75_Mn_0.25_‐LDH precursor (Figure S3, Supporting Information), peaks at 780.3 and 797.1 eV are assigned to Co^2+^ 2p_3/2_ and 2p_1/2_, peaks at 712.3 and 724.6 eV are attributed to Fe^3+^ 2p_3/2_ and 2p_1/2_, and peaks at 642.2 and 653.8 eV correspond to Mn^4+^ 2p_3/2_ and 2p_1/2_. After vulcanization (Figure S4, Supporting Information), the Co 2p_3/2_ and 2p_1/2_ binding energies of 779.5 and 794.2 eV are characteristic of CoS_2_. The Fe 2p_3/2_ peaks at 707.8, 710.3 eV and Fe 2p_1/2_ band at 720.6 eV can be assigned to Fe^2+^. The relative proportions of Fe^2+^ and Fe^3+^ were determined to be 67.2% and 32.8%. The peaks at 641.0, 642.0, and 644.0 eV are attributed to Mn^2+^, Mn^3+^ and Mn^4+^ with proportions of 18.3%, 55.0%, and 26.7% respectively. In the S 2p region, the peaks at 162.6 and 163.9 eV are attributed to S^2−^, while the minor peak at 167.9 eV can be ascribed to the presence of a small amount of SO_4_^2−^.

The physicochemical properties of the Co_2_Fe_0.75_Mn_0.25_S_6_ and Co_2_Fe_0.75_Mn_0.25_S_6_‐PVP NSs were further investigated. CFMS NSs show a negative zeta potential of −14.7 ± 0.2 mV in aqueous suspension, in contrast to the positive potential of the LDH sample (21.0 ± 0.7 mV, Figure 1J). After PVP functionalization, the zeta potential of the CFMS‐PVP NSs changed to −20.2 ± 0.3 mV, −19.7 ± 0.2 and −19.4 ± 0.1 mV in aqueous solution, phosphate buffer saline (PBS) and pure Dulbecco's Modified Eagle's Medium (DMEM). The CFMS‐PVP NSs showed a lateral size of 57.0 ± 4.3 nm and a thickness of ≈3.2 nm (Figures S5 and S6, Supporting Information). Dynamic light scattering (DLS) data for CFMS‐PVP NSs reveal the hydrodynamic particle size to be around 101.5 ± 5.5 nm in three different media (water, PBS, and DMEM), and there are no notable changes in particle diameter distribution over 7 days of storage (Figure 1K,L). An obvious Tyndall effect can be seen for CFMS‐PVP NSs dispersed in water, DMEM and PBS during storage for 7 days (Figure S7, Supporting Information), which demonstrates their satisfactory storage stability and good dispersibility.

The morphology and size distributions of CFMS‐PVP samples prepared from a range of different LDH precursors with varied metal ratios were also explored. TEM and DLS both show a lamellar morphology with hydrodynamic particle size of ≈100.0 nm (Figure S8, Supporting Information). The materials are hence all similar in terms of particle size and shape regardless of the metal composition. The mass ratio of PVP in the final Co_2_Fe_0.75_Mn_0.25_S_6_‐PVP NSs was estimated to be 6.40% by thermogravimetry (Figure S9, Supporting Information).

### 2.2 Photothermal and Chemodynamic Properties of CFMS‐PVP NSs

**Figure 2:**
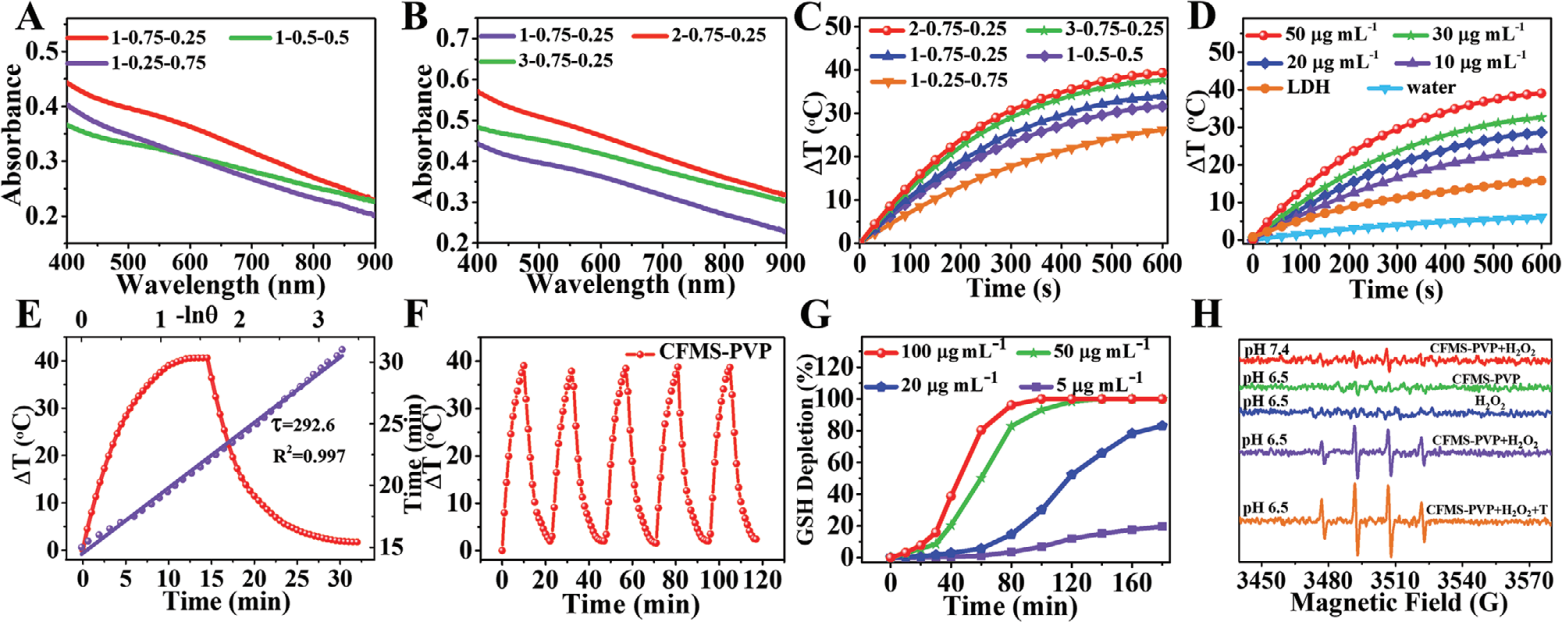
PTT and CDT performance. A,B) UV–vis absorbance spectra and C) photothermal heating curves of CFMS‐PVP NSs with different ratios of Co: Fe: Mn. D) Temperature elevation of CFMS‐PVP NS suspensions (Co: Fe: Mn = 2: 0.75: 0.25) at different concentration (0, 10, 20, 30, and 50 µg mL^−1^) and the Co_2_Fe_0.75_Mn_0.25_‐LDH precursor (50 µg mL^−1^) upon 808 nm laser irradiation (1.0 W cm^−2^). E) Calculation of the PCE at 808 nm. The time constant (*τ*_s_) for the heat transfer was calculated from the cooling period (purple line). F) Temperature variation of Co_2_Fe_0.75_Mn_0.25_S_6_‐PVP NSs under irradiation at 1.0 W cm^−2^ for 5 on/off cycles (10 min irradiation for each cycle). G) GSH depletion under reduction by Co_2_Fe_0.75_Mn_0.25_S_6_‐PVP NSs. H) ESR spectra of Co_2_Fe_0.75_Mn_0.25_S_6_‐PVP NSs with H_2_O_2_ at different pH and temperatures (spectra of the NSs and H_2_O_2_ alone are shown as controls).

The influence of the Co/Fe/Mn molar ratio on the crystal structure and PTT performance of CFMS‐PVP NSs was studied. Through altering the ratio of Fe/Mn (0.25: 0.75, 0.5: 0.5, and 0.75: 0.25) in the LDH precursor, with constant Co content, a series of TMDs could be obtained. The crystallinity of the CFMS NSs changes markedly, and a ratio of 0.75: 0.25 gives rise to the strongest reflection intensities in XRD (Figure S2A, Supporting Information). With an increased content of Co, metal–ligand charge transfers from Co 3d to S 4s orbitals and d–d transitions of Co will enhance absorption in the UV–vis region.^[^ ^14^ ^]^ However, when altering the ratio of Co (from 1 to 3) with constant Fe/Mn (0.75: 0.25), the reflection intensity of the CoS_2_/FeS_2_ phase declines markedly (Figure S2B, Supporting Information), and only Co_1_Fe_0.75_Mn_0.25_S_4_ and Co_2_Fe_0.75_Mn_0.25_S_6_ retain the CoS_2_/FeS_2_ structure. The near‐infrared (NIR) absorption of the various CFMS‐PVP NSs (Co*_x_*Fe*_y_*Mn_1−_ *_y_*S_2(_ *_x_*_+1)_‐PVP) was recorded, and the Co_2_Fe_0.75_Mn_0.25_S_6_‐PVP sample showed the greatest absorbance at 808 nm (**Figure** **2**A,B). Results from inductively coupled plasma (ICP) show that the molar ratio of Co/Fe/Mn in the CFMS products is close to the feed ratio (Table S1, Supporting Information).

Subsequently, the Co*_x_*Fe*_y_*Mn_1−_ *_y_*S_2(_ *_x_*_+1)_‐PVP samples were irradiated with an 808 nm laser at a power density of 1.0 W cm^−2^, and the temperature changes were monitored. As shown in Figure 2C, Co_2_Fe_0.75_Mn_0.25_S_6_‐PVP NSs possess the best photothermal performance, which is consistent with the NIR absorption data. The photothermal performance of the Co_2_Fe_0.75_Mn_0.25_S_6_‐PVP NSs is strongly dependent on the concentration and the laser power: an increase in temperature (Δ*T*) ranging from 24.7 to 39.4 °C is obtained along with the increase of concentration (10−50 µg mL^−1^, Figure 2D), and Δ*T* can be finely tuned from 9.7 to 39.4 °C by enhancing the laser power (0.25−1.0 W cm^−2^, Figure S10, Supporting Information). A clear visual change in temperature can be seen for CFMS suspensions when using a thermal infrared imaging device (Figure S11, Supporting Information).

In contrast, control samples (PBS and CoFeMn‐LDH suspension: 50 µg mL^−1^) exposed to the NIR laser only give a temperature increment of 6.0 and 15.8 °C, respectively. Notably, the PCE of Co_2_Fe_0.75_Mn_0.25_S_6_‐PVP NSs is determined to be 89.0% (Figure 2E), significantly greater than for previously reported 2D TMDs such as TaS_2_‐PEG NSs (39.0%), Cu‐Fe‐Se NSs (78.9%), ReS_2_ NSs (79.2%), etc. (Table S2, Supporting Information). We further compared the photothermal effect of Co_2_Fe_1_S_6_‐PVP and the Co_2_Fe_0.75_Mn_0.25_S_6_‐PVP NSs. Co_2_Fe_1_S_6_‐PVP NSs leads to an increase of 31.4 °C while CFMS‐PVP NSs induces a 39.4 °C increment after 10 min irradiation at the power density of 1.0 W cm^−2^. The PCE (*η*) values for Co_2_Fe_0.75_Mn_0.25_S_6_‐PVP and Co_2_Fe_1_S_6_‐PVP NSs are 89.0% and 76.4% respectively (Figure S12, Supporting Information), demonstrating the superior photothermal performance of the CFMS‐PVP NSs. The high PCE of the Co_2_Fe_0.75_Mn_0.25_S_6_‐PVP NSs can be attributed to the following factors. Firstly, the ultrathin 2D structure of the CFMS‐PVP NSs endows them with a large surface area (185.59 m^2^ g^−1^; Figure S13, Supporting Information). This permits them to serve as laser‐cavity mirrors with improved absorption of photoelectrons, leading the interaction time between the photoelectrons and the material being extended. Secondly, nanomaterials with smaller bandgaps possess higher PCE in comparison with those with wider bandgaps.^[^ ^15^ ^]^ The bandgap of the Co_2_Fe_0.75_Mn_0.25_S_6_‐PVP NSs was determined to be 0.57 eV, highly beneficial for PCE (Figure S14, Supporting Information). Thirdly, compared with two‐metal materials, multi‐metal coordination endows TMDs with an enhanced PCE. This is clear from a comparison of Co_2_Fe_1_S_6_‐PVP and the Co_2_Fe_0.75_Mn_0.25_S_6_‐PVP NSs, where the latter show noticeably better photothermal performance (Figure S12, Supporting Information). In addition, no significant deterioration in performance can be detected after 5 successive heating‐cooling cycles (Figure 2F), confirming the photothermal stability of the CFMS‐PVP NSs.

Inspired by the excellent photothermal conversion properties of the Co_2_Fe_0.75_Mn_0.25_S_6_‐PVP NSs, their photoacoustic properties were evaluated.^[^ ^16^ ^]^ A significant enhancement of PA signal intensity is observed for CFMS‐PVP NSs with an increase of sample concentration, with a linear correlation from 0 to 50 µg mL^−1^ (Figure S15, Supporting Information). The PA signal intensity from a suspension of the CFMS‐PVP NSs was about 3.3 times higher than an LDH suspension at the same concentration, indicating a markedly enhanced PA performance after vulcanization.

GSH can react with the ·OH generated in CDT, and oxidation of intracellular GSH to GSSG is highly beneficial for effective CDT treatment. We thus investigated the reaction between CFMS‐PVP NSs and GSH. 5, 5‐Dithiobis‐(2‐nitrobenzoic acid (DTNB) was used to detect GSH depletion after the addition of different concentrations of Co_2_Fe_0.75_Mn_0.25_S_6_‐PVP NSs.^[^ ^17^ ^]^ As shown in Figure 2G, significantly increased GSH depletion (from an initial concentration of 1.0 × 10^−3^ m) was observed with increasing CFMS‐PVP NS concentrations from 5 to 100 µg mL^−1^, which verified that the NSs are effective for GSH depletion. Electron spin resonance (ESR) spectroscopy was applied to detect ·OH generated in situ, using 5, 5‐dimethyl‐1‐pyrroline‐N‐oxide (DMPO) as the probe (Figure 2H). ESR signals could barely be observed for Co_2_Fe_0.75_Mn_0.25_S_6_‐PVP NSs + H_2_O_2_ at neutral pH conditions (pH 7.4), while increased ESR signal intensity was noted in mildly acidic conditions (pH 6.5). This reveals specificity of the CFMS‐PVP NSs, in terms of their generating radicals only in the mildly acidic tumor microenvironment. Much more intense ·OH peaks can be detected at elevated temperature (318 K), suggesting an increased temperature accelerates the Fenton reaction and generates larger amounts of ·OH. After reaction with GSH, the Co_2_Fe_0.75_Mn_0.25_S_6_‐PVP NSs retain their lamellar morphology. However, partial decomposition of the NSs can be observed in terms of a reduced hydrodynamic particle size (75.7 ± 8.7 nm, Figure S16, Supporting Information). XPS was utilized to study the change in valence state of Mn, Co and Fe in CFMS‐PVP NSs after treatment with GSH. The oxidation states of Co and Fe remain unchanged, but most of Mn^3+^/Mn^4+^ has been reduced to Mn^2+^. This is clear from the presence of a typical Mn^2+^ satellite peak (≈647.0 eV), which is absent for either Mn^3+^ or Mn^4+^ (Figure S17, Supporting Information).

To further investigate the occurrence of Fenton reactions, 3, 3, 5, 5‐tetramethylbenzidine (TMB) was employed for a kinetic analysis.^[^ ^18^ ^]^ The changes in absorbance occurring upon the addition of H_2_O_2_ (0.05, 0.1, 0.2, 0.5, 1.0, and 2.0 × 10^−3^ m) to a suspension of Co_2_Fe_0.75_Mn_0.25_S_6_‐PVP NSs (50 µg mL^−1^) are depicted in Figure S18 (Supporting Information). The corresponding initial velocities (*V*_0_) were calculated via the Beer–Lambert law. A range of initial velocities were then fitted with the Michaelis–Menten equation and a linear double reciprocal plot (Lineweaver–Burk plot) constructed to obtain the Michaelis–Menten constant (*K*_m_) and maximum velocity (*V*_max_) (experimental details and the calculation procedure are given in the Experimental Section). The *K*_m_ and *V*_max_ values were calculated to be 0.46 × 10^−3^ m and 2.74 × 10^−8^ m s^−1^ for the CFMS‐PVP NSs at 298 K (Figure S18A–C, Supporting Information). When the temperature was increased temperature to 318 K, the *K*_m_ and *V*_max_ values obtained are 0.26 × 10^−3^ m and 3.77 × 10^−8^ m s^−1^ (Figure S18D–F, Supporting Information), confirming that heat produced from the CFMS‐PVP NSs could accelerate the generation of ·OH.

### 2.3 In Vitro Biocompatibility and PTT/CDT Study

**Figure 3:**
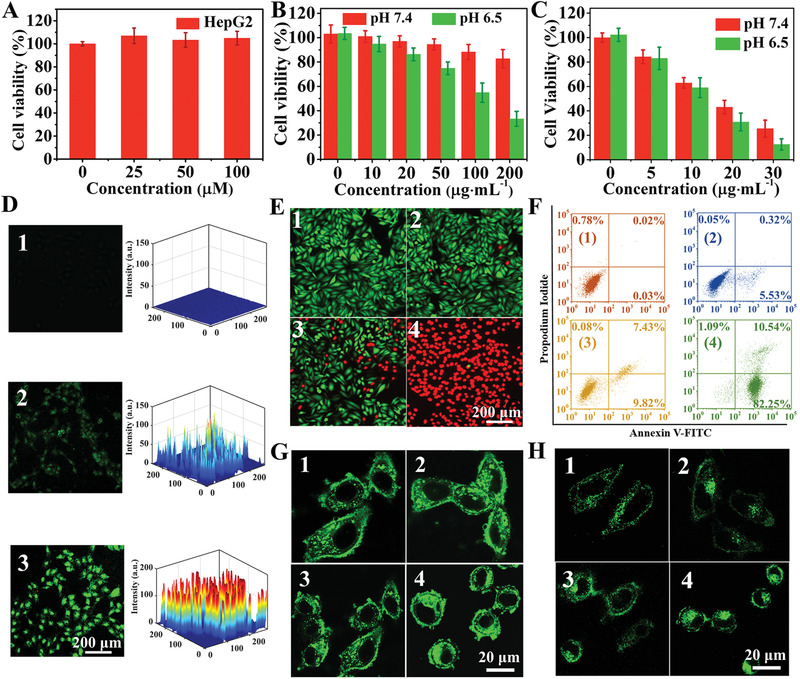
In vitro PTT/CDT studies with HepG2 cells. Relative viabilities of HepG2 cells are shown after incubation A) with different concentrations of H_2_O_2_, and B,C) with Co_2_Fe_0.75_Mn_0.25_S_6_‐PVP NSs and H_2_O_2_ (100 × 10^−6^ m) at different pH values, with (B) and without (C) 808 nm laser irradiation at 1.0 W cm^−2^ for 8 min. D) Confocal images of DCFH‐DA (2, 7‐dichlorofluorescein diacetate) stained cells. E) Viability of cells in the presence of the CFMS‐PVP NSs. F) Cell apoptosis analysis using the Annexin V‐FITC/PI double staining method: 1) PBS; 2) pH 7.4 + H_2_O_2_ (100 × 10^−6^ m); 3) pH 6.5 + H_2_O_2_ (100 × 10^−6^ m); 4) pH 6.5 + H_2_O_2_ (100 × 10^−6^ m) + NIR (1.0 W cm^−2^, 8 min). Confocal laser scanning microscope (CLSM) images of (G) LysoTracker Green DND‐26 (green) stained lysosomes and H) MitoTracker Green FM (green) stained mitochondria. Numbered panels correspond to the following treatments: 1) PBS; 2) pH 7.4 + H_2_O_2_ (100 × 10^−6^ m); 3) pH 6.5 + H_2_O_2_ (100 × 10^−6^ m); 4) pH 6.5 + H_2_O_2_ (100 × 10^−6^ m) + NIR (1.0 W cm^−2^, 8 min).

Prior to investigating the anticancer effect of the Co_2_Fe_0.75_Mn_0.25_S_6_‐PVP NSs, a biocompatibility evaluation was conducted on HepG2, HeLa, and U87mg cell lines. No obvious toxicity was observed even at a concentration up to 200 µg mL^−1^, which indicates the CFMS‐PVP NSs have good biocompatibility (Figure S19, Supporting Information). The survival rate of HepG2 cells also remains unchanged upon the addition of H_2_O_2_, even at 100 × 10^−6^ m (**Figure** **3**A). Subsequently, in vitro CDT induced by CFMS‐PVP NSs was investigated. After incubation with 200 µg mL^−1^ of CFMS‐PVP NSs and 100 × 10^−6^ m H_2_O_2_ for 24 h, cell viabilities of 32.7% and 82.7% were obtained at pH 6.5 and pH 7.4 respectively (Figure 3B).

To explore the ability of the Co_2_Fe_0.75_Mn_0.25_S_6_‐PVP NSs in combined PTT/CDT, HepG2 cells were incubated with the NSs (at 5–30 µg mL^−1^) for 24 h and then irradiated with a 808 nm laser (1.0 W cm^−2^) for 8 min. Without H_2_O_2_, the PTT efficacy of CFMS‐PVP NSs was investigated, and the cell viability of 29.8% was obtained at the concentration of 30 µg mL^−1^ (Figure S20, Supporting information). In the presence of 30 µg mL^−1^ of Co_2_Fe_0.75_Mn_0.25_S_6_‐PVP NSs and H_2_O_2_ (100 × 10^−6^ m), cell viability was 12.5% and 25.4% at pH 6.5 and pH 7.4, indicating a synergistic effect from PTT/CDT (Figure 3C). To further probe the synergistic effects, the half‐maximal inhibitory concentrations (IC_50_) of CFMS‐PVP NSs was calculated to obtain the combination index (CI).^[^ ^19^ ^]^ The CI value is less than 1.0, indicating an excellent synergistic effect of PTT/CDT.

The production of ·OH in the HepG2 cellular environment was proven using the intracellular ROS probe 2, 7‐dichlorofluorescein diacetate (DCFH‐DA). Compared with the control group, HepG2 cells incubated with CFMS‐PVP NSs give a weak green fluorescence signal at pH 7.4. Notably enhanced green emission was observed at pH 6.5, demonstrating that a large amount of intracellular ·OH was produced (Figure 3D). Moreover, HepG2 cells treated with saline, and Co_2_Fe_0.75_Mn_0.25_S_6_‐PVP NSs with 808 nm irradiation were stained with calcein‐AM and propidium iodide (PI) (Figure 3E). The results concur with the cytotoxicity data above. When the treated HepG2 cells were stained with Annexin V‐FITC/PI and analyzed by flow cytometry to monitor cell apoptosis,^[^ ^20^ ^]^ 92.8% apoptosis was observed in HepG2 cells treated with CFMS‐PVP NSs and exposed to 8 min of irradiation. Significant early‐stage apoptosis appeared in the PI^+^/Annexin V‐FITC^+^ region, demonstrating effective PTT/CDT is induced by the CFMS‐PVP NSs (Figure 3F).

Cell apoptosis usually originates from injury of subcellular organelle structures, including lysosomes, mitochondria, etc.^[^ ^21^ ^]^ We thus examined the influence of PTT/CDT on the lysosomes (Figure 3G). Compared with untreated groups and cells exposed to CFMS‐PVP NSs alone, the green spotted lysosomes in the cells partially accumulated in the cytoplasm and the morphology of the cells changed somewhat in the presence of Co_2_Fe_0.75_Mn_0.25_S_6_‐PVP NSs and H_2_O_2_ at pH 6.5. After exposure to the 808 nm laser, the green spots accumulated and the intensity of the fluorescence signal increased in almost the entire cytosol, implying that the CFMS‐PVP NSs can destroy lysosomes and result in lysosomal membrane permeabilization. We further explored the effects of the different treatments on the mitochondria (Figure 3H). When cells were treated with CFMS‐PVP NSs and H_2_O_2_ at pH 6.5, the mitochondria became partially fragmented due to ·OH production. Mitochondrial fragmentation increased greatly after Co_2_Fe_0.75_Mn_0.25_S_6_‐PVP NSs incubation plus laser irradiation, demonstrating severe damage to mitochondria. It is concluded that local hyperthermia and ·OH from CFMS‐PVP NSs induce mitochondrial dysfunction and contribute to apoptosis.

### 2.4 In Vivo Anti‐Tumor Therapy

**Figure 4:**
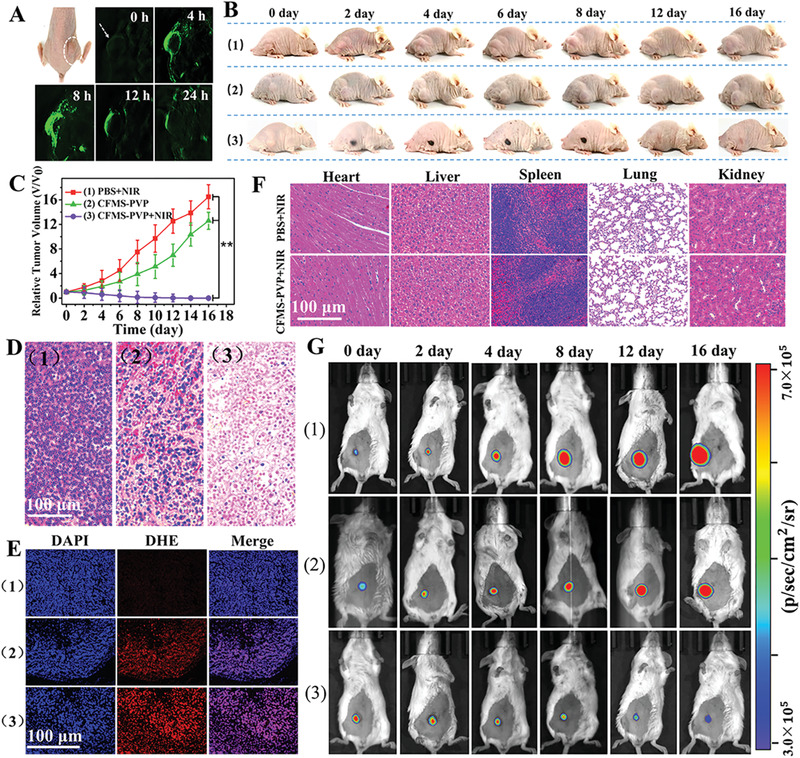
In vivo results in tumor‐bearing mice. Treatment groups are: 1) PBS + NIR, 2) CFMS‐PVP NSs, 3) CFMS‐PVP NSs + NIR. A) Multispectral optical tomography system (MSOT) images of tumors tissue (arrows) at different time points after injection via the tail vein. B) Digital photographs. C) Relative tumor volumes (*n* = 6, mean ± S.D). D) H&E‐stained histological images of the tumor site. E) H&E‐stained histological images of the major organs. F) Fluorescence images of tumor slices after DHE staining (red). The nuclei were stained with DAPI (blue). G) Bioluminescence imaging of 4T1‐Fluc‐tumor‐bearing mice. *P* values were calculated by ANOVA followed by Tukey's post‐test (*P < 0.05, **P < 0.01).

Encouraged by the in vitro results, therapeutic efficacy in vivo was evaluated in HepG2 tumor‐bearing mice via intravenous (i.v.) injections (200 µL, 10 mg kg^−1^). We first assessed the PA performance of Co_2_Fe_0.75_Mn_0.25_S_6_‐PVP NSs in the HepG2 tumor‐bearing model (*n* = 3 mice/group). As shown in **Figure** **4**A, no PA signal was found in the tumor area (white arrow) before injection, but a distinct PA signal clearly distinguishable from the background began to appear after 4 h and reached a peak 8 h after i.v. injection. The PA signal then decreased rapidly and became undetectable 24 h post‐injection.

Subsequently, mice were randomly divided into three groups: 1) PBS + NIR (6 min irradiation, control group), 2) CFMS‐PVP NSs (CDT alone), 3) CFMS‐PVP NSs + NIR (6 min irradiation, PTT/CDT). 8 h after i.v. administration, in groups (1) and (3) the mice were anesthetized and then exposed to a 808 nm laser (1.0 W cm^−2^). In vivo photothermal images to visualize temperature changes were recorded using a thermal infrared imaging device (Figure S21, Supporting Information). Compared with the PBS group, the tumor‐site temperature of mice injected with Co_2_Fe_0.75_Mn_0.25_S_6_‐PVP NSs increased rapidly from 34.3 to 54.2 °C over 6 min (Figure S22, Supporting Information). Digital photos and the tumor volume were recorded every other day for 16 days after PTT/CDT treatment. The PBS groups show rapid tumor growth, while treatment with Co_2_Fe_0.75_Mn_0.25_S_6_‐PVP NSs but without NIR irradiation partially inhibits the tumor growth as a result of CDT. For mice receiving both CFMS‐PVP NSs and irradiation, the tumors were completely ablated during the experimental period (Figure 4B,C). Figure S23 (Supporting Information) displays the excised tumors obtained when the mice were sacrificed after 16 days. These are consistent with the tumor volume curves measured in vivo.

Tumor tissue slices collected from three groups were stained with hematoxylin and eosin (H&E) (Figure 4D). Very clear necrosis of the HepG2 cells was observed in the Co_2_Fe_0.75_Mn_0.25_S_6_‐PVP NSs + NIR group, while the PBS + NIR group retained a normal cell morphology and the CFMS‐PVP NSs group showed partial necrosis. To verify ·OH production in tumors as a result of the presence of the CFMS‐PVP NSs, DHE was used to detect its generation in vivo. No obvious red fluorescence was observed with PBS + NIR, while red fluorescence was visible in the CFMS‐PVP NSs group. The fluorescence intensity became stronger when the NSs were administered with NIR irradiation, confirming photothermal‐enhanced CDT (Figure 4E).

The Co concentrations in the major organs and tumor sites were measured by ICP to investigate in vivo accumulation and metabolism of the CFMS‐PVP NSs (Figure S24A, Supporting Information). A high level of Co was observed at the tumor site as well as in most organs in the first 8 h, but then declined gradually to a low level at 48 h, demonstrating efficient degradation or excretion of the NSs. A high level of Co was detected in both urine and feces, especially in the first 8 h (Figure S24B, Supporting Information), which indicates the CFMS‐PVP NSs can be excreted through the liver and kidney. After 48 h, again the Co concentrations had markedly declined. These results indicate good in vivo biocompatibility, which was further supported by H&E staining analysis of the major organs (i.e., heart, liver, spleen, lung, kidney; Figure 4F). No significant differences are observed between the CFMS‐PVP NSs and PBS groups. Furthermore, no change in body‐weight is seen throughout the experimental period (Figure S25, Supporting Information), indicating that all treatments had negligible off‐target side effects. After mice were treated with the CFMS‐PVP NSs, their blood biochemistry indices (WBC, RBC, PLT, and HGB) and levels of liver and kidney function indices (AST, ALT, BUN and CRE) did not differ from those recorded with healthy mice, again consistent with there being negligible side effect of CFMS‐PVP NSs administration (Figure S26, Supporting Information).

A second in situ tumor model was developed using 4T1 tumor cells labeled with firefly luciferase. Bioluminescence imaging was used to investigate the antitumor performance of CFMS‐PVP NSs through the specific binding of firefly luciferase and fluorescein. This allows rapid, sensitive and noninvasive in vivo detection as well as quantitative analysis of tumor growth and metastasis.^[^ ^22^ ^]^ As shown in Figure 4G and Figure S27 (Supporting Information), tumors grew rapidly in the PBS control group, while treatment with CFMS‐PVP NSs partly suppressed growth. In contrast, application of CFMS‐PVP NSs and exposure to 808 nm laser irradiation led to complete tumor destruction and atrophy. These results all confirm that the CFMS‐PVP NSs have excellent and synergistic PTT/CDT performance, and thus potential clinical applications.

## 3 Conclusion

A highly tunable CFMS‐PVP NSs platform was fabricated based on the vulcanization of a lamellar CoFeMn‐LDH precursor. The NSs allow effective PA imaging and synergistic PTT/CDT therapy. Outstanding therapeutic outcomes are observed in vivo, arising from the excellent PCE (*η* = 89.0%) and multi‐functionality of the CFMS‐PVP NSs. The presence of Co and Fe causes conversion of H_2_O_2_ overexpressed in tumor cells into toxic ·OH, and the Mn reduces intracellular GSH. The temperature rise elicited by NIR laser irradiation further accelerated the disproportionation of H_2_O_2_, giving PTT‐enhanced CDT. Both in vitro and in vivo therapeutic efficacy tests demonstrate inhibition of HepG2 cell proliferation, and complete eradication of tumors can be caused by the combined PTT/CDT effects of the CFMS‐PVP NSs. Therefore, the ternary‐metal CFMS‐PVP NSs synthesized here hold great promise for future clinical cancer theranostics.

## 4 Experimental Section

##### Materials

Cobalt nitrate (Co(NO_3_)_2_·6H_2_O), iron nitrate (Fe(NO_3_)_3_·9H_2_O), magnesium nitrate (Mn(NO_3_)_2_, 50% W/W), sodium hydroxide (NaOH), sodium nitrate (NaNO_3_), formamide, hydrogen peroxide solution (H_2_O_2_, 30%), 5, 5‐dithiobis‐(2‐nitrobenzoic acid) (DTNB), polyvinyl pyrrolidone (PVP, MW = 10 000), and thioacetamide (TAA) were purchased from Aladdin Chemical. Co. Ltd (Shanghai, China). 2, 7‐dichlorodihydrofluorescein diacetate (DCFH‐DA), calceinacetoxymethyl ester (Calcein‐AM) and propidium iodide (PI) were purchased from Sigma‐Aldrich (St. Louis, MO, USA). Dulbecco's modified Eagles medium (DMEM), 0.25% trypsin‐EDTA and penicillin/streptomycin were obtained from Gibco (Invitrogen, Carlsbad, CA). Fetal bovine serum (FBS) was purchased from Excell Bio. Co., Ltd. (Shanghai, China). An Annexin V‐FITC & propidium iodide apoptosis detection kit was purchased from Solarbio Science and Technology Co, Ltd (Beijing, China). Mito‐Tracker Green and Lyso‐Tracker Green were purchased from Beyotime (Beijing, China). Ultrapure water from a Milli‐Q Millipore system was used in all processes. All chemicals were of analytical‐grade and obtained from commercial sources, and were used without any further purification.

##### Preparation of CoFeMn‐LDH Precursor

The synthesis of CoFeMn‐LDH precursor with varying Co*_x_*Mn*_y_*Fe_1−_ *_y_* was performed using a bottom‐up method previously reported by the group, with some modifications. In a typical procedure, solution A was prepared from Co(NO_3_)_2_·6H_2_O (0.4 mmol), Fe(NO_3_)_3_·9H_2_O (0.15 mmol), and Mn(NO_3_)_2_ (0.05 mmol) in deionized water (20 mL). Solution B comprised NaNO_3_ (0.1 mmol) dissolved in deionized water (20 mL) containing 25% v/v formamide. A third solution, solution C was prepared by dissolving NaOH (0.0025 mmol) in deionized water (20 mL). Solution A and solution C were then added simultaneously into solution B, with stirring at the speed of 600 rpm at 80 °C. After cooling to ambient temperature, the products were collected by centrifugation at 7000 rpm, then the samples were washed with ethanol and deionized water three times followed by dialysis (3 kDa) to remove formamide.

##### Preparation of CFMS NSs

The CFMS NSs were prepared using the CoFeMn‐LDH precursor and thioacetamide (TAA). Briefly, TAA (0.12 mol) was first dissolved in ethanol (40 mL) in a Teflon container (100 mL), followed by the addition of the CoFeMn‐LDH precursor (0.06 mol). After hydrothermal treatment at 120 °C for 12 h, the product was collected by centrifugation at 7000 rpm for 5 min and washed thoroughly with water and ethanol. The CFMS NSs were finally re‐dispersed in deionized water for subsequent experiments.

##### Preparation of CFMS‐PVP NSs

PVP (0.06 mol) was added into the CFMS solution with ultrasonication for 30 min and then stirred at 600 rpm for 8 h. The CFMS‐PVP NSs were collected by centrifugation at 7000 rpm for 5 min and washed thoroughly with deionized water and ethanol three times.

##### Measurement of Photothermal Performance

CFMS‐PVP NSs (10, 20, 30, and 50 µg mL^−1^), LDH suspension (50 µg mL^−1^) and pure water were individually placed in a quartz cuvette and then irradiated with a 808 nm NIR laser (1.0 W cm^−2^) for 10 min. The temperature changes and thermal infrared images were measured by a thermal infrared imaging device (Ti450, Fluke, Everett, WA, USA). The PCE (*η*) can be determined from Equation (1)

(1) $$\eta =\frac{hs\left(T_{\max}-T_{\mathrm{surr}}\right)-Q_{\mathrm{dis}}}{I\left(1-10^{\left(-A_{\lambda }\right)}\right)}$$

Where *h* represents the heat transfer coefficient of the CFMS‐PVP NSs suspension, *s* is the surface area of the container, *T*_max_ is the maximum temperature of the suspension, *T*_Surr_ is the temperature of the surroundings, *Q*_dis_ is the heat associated with light absorbance by the solvent, *I* is the incident laser power (1.0 W cm^−2^), and *A*_808_ is the absorbance of the CFMS‐PVP NSs at 808 nm.

To get the value of *hs*, *θ* is expressed using the maximum system temperature

(2) $$\theta =\frac{T-T_{\mathrm{surr}}}{T_{\max}-T_{\mathrm{surr}}}$$

*τ*_s_ can be derived according to Equation (3)

(3) $$t=-\tau_{s}ln\theta$$

Then, *hs* can be determined from Equation (4)

(4) $$hs=\frac{m_{D}C_{D}}{\tau_{s}}$$

where *τ*_s_ is the time constant for the heat transfer, and *m*_D_ and *C*_D_ are the mass (1.0 g) and heat capacity (4.2 J g^−1^) of deionized water used to disperse the CFMS‐PVP NSs.

*Q*_dis_ represents the heat input absorbed by pure water and the quartz cuvette, and can be described as follows

(5) $$Q_{\mathrm{dis}}=\frac{m_{D}C_{D}\left(T_{\max\left(\mathrm{water}\right)}-T_{\mathrm{surr}}\right)}{\tau_{\mathrm{water}}}$$

Where *T*_max (water)_ is 34.6 °C, *T*_surr_ is 28.6 °C, *τ*_water_ is 356. Hence, *Q*_dis_ was calculated to be 0.071 W. Therefore, *τ*_s_ for heat transfer from the CFMS‐PVP NSs was determined to be 292.6 s by fitting a straight line to the data from the cooling period versus −ln *θ*. Thus, according to Equation (4), *hs* was determined to be 14.35 mW °C^−1^. Finally, the PCE (*η*) of the CFMS‐PVP NSs can be calculated to be 89.0%.

##### Extracellular Depletion of GSH

The consumption of GSH was monitored by absorption in the DTNB assay. Solutions containing 1 × 10^−3^ m GSH were added to different amounts of CFMS‐PVP NSs (5, 20, 50, and 100 µg mL^−1^). The solutions (4 mL) were incubated for different times (0, 10, 20, 40, 60, 80, 100, 120, and 140 min) and then centrifuged at 10 000 rpm for 10 min to remove the CFMS‐PVP NSs. After 10 min of coincubation of the supernatant (2 mL) and DTNB solution (0.8 × 10^−3^ m, 2 mL), absorbance was recorded using a microplate reader.

##### OH Production

5, 5‐dimethyl‐1‐pyrroline N‐oxide (DMPO) was used as a ·OH trapping agent. Reaction groups were established as follows: 1) 20 µL H_2_O_2_ (5 × 10^−3^ m) + 1 µg CFMS‐PVP NSs at pH 7.4; 2) 20 µL H_2_O_2_ at pH 6.5; 3) 1 µg CFMS‐PVP NSs at pH 6.5; 4) 20 µL H_2_O_2_ + 1 µg CFMS‐PVP NSs at pH 6.5, and 5) 20 µL H_2_O_2_ + 1 µg CFMS‐PVP NSs with 5 min heating using 318 K water bath at pH 6.5. Subsequently, 400 µL of DMPO buffer solution (100 × 10^−3^ m) and each reaction mixture were added to a quartz capillary, and X‐band EPR spectra were measured in perpendicular mode on a Bruker (Germany) EMX‐500 10/12 spectrometer with the following settings: microwave frequency = 9.872 GHz, microwave power = 6.375 mW, modulation frequency = 100.00 kHz and modulation amplitude = 1.00 G.

##### Michaelis–Menten Kinetics

The Fenton performance of CFMS‐PVP NSs was studied by monitoring the absorption spectra of TMB at 652 nm using a microplate reader. In a typical test, CFMS‐PVP NSs (final concentration 50 µg mL^−1^), TMB (final concentration 800 × 10^−6^ m) and varied concentrations of H_2_O_2_ (0.05, 0.10, 0.20, 0.50, 1.0, and 2.0 × 10^−3^ m) were suspended in a Acetic acid‐Sodium acetate (HAc‐NaAc) buffer solution (pH 6.5) at controlled temperature (298 and 318 K). Then Michaelis–Menten constant was calculated according to Lineweaver–Burk plots, based on Equations (6) to (8).

(6) A=Kbc

(7) $$v_{0}=\frac{V_{\max}\left[S\right]}{K_{M}+\left[S\right]}$$

(8) $$\frac{1}{v_{0}}=\frac{K_{M}}{V_{\max}}\frac{1}{\left[S\right]}+\frac{1}{V_{\max}}$$

Where *A* is absorbance of TMB at 652 nm, *K* represents the molar absorption coefficient, *b* is the thickness of the container, *c* is the concentration of TMB, *v*_0_ is the initial velocity, *V*_max_ represents the maximum reaction velocity, *S* is the substrate concentration, and *K*_m_ expresses the Michael constant.

In Vitro *PA Imaging*: In vitro PA imaging was performed on a multispectral optical tomography system (MSOT inVision 256, iThera 8 Medical, Germany). CFMS‐PVP NSs aqueous suspension at different concentrations (2.5, 5, 10, 20, and 50 µg mL^−1^, 1 mL) were placed in agar gel cylinders for in vitro PA imaging.

##### Cell Culture

HepG2, U87mg, and HeLa cell lines obtained from Basic Medical Sciences Chinese Academy of Medical Sciences (Beijing, China) were cultured in DMEM supplemented with 10% v/v fetal bovine serum (FBS), 1% streptomycin and penicillin (v/v, Corning, USA) under a humidified atmosphere of 5% CO_2_ at 37 °C.

##### In Vitro Cytotoxicity

For cell viability studies, HepG2, U87mg, and Hela cells were seeded in 96‐well plates (1 × 10^4^ cells per well, in 200 µL of medium) for 24 h. Then the culture medium was replaced by fresh culture medium containing CFMS‐PVP NSs at different concentrations (0, 10, 20, 50, 100, 200 µg mL^−1^, 200 µL). After incubation of 24 h and thorough washing with PBS, the cell viability was determined by the 3‐[4, 5‐dimethylthiazol‐2‐yl]‐2, 5‐diphenyltetrazolium bromide (MTT) assay.

##### In Vitro CDT Measurement

HepG2 cells were seeded in 96‐well plates (1 × 10^4^ cells per well, 200 µL) for 24 h. The pH of the media was adjusted (7.4 and 6.5) to simulate the extracellular microenvironment in a solid tumor. HepG2 cells were incubated with fresh medium (pH 7.4 and pH 6.5) containing 100 × 10^−6^ m H_2_O_2_ and CFMS‐PVP NSs at concentrations of 10, 20, 30, 50, 100, and 200 µg mL^−1^ (200 µL per well) for another 24 h. The cell viability was measured by the MTT assay.

##### In Vitro Synergistic CDT and PTT

HepG2 cells were seeded in 96‐well plates at a density of 1 × 10^4^ cells per well (200 µL) for 24h. Then the culture medium was replaced by fresh culture medium containing CFMS‐PVP NSs at different concentrations (2.5, 5, 10, 20, and 30 µg mL^−1^, 200 µL per well) at the same time, and further co‐incubated for 12 h, cells were irradiated with 808 nm NIR light (1.0 W cm^−2^) for 10 min. The MTT assay was used to evaluate the cytotoxicity of the various treatments after another 12 h. To verify the MTT results, Calcein‐AM/PI was used to stain living and dead cells according to the manufacturer's instructions.

##### Detection of Intracellular ·OH Production

The DCFH‐DA fluorescent probe was used to detect intracellular ·OH. HepG2 cells were seeded into 12‐well culture dishes (1 × 10^5^ cells per well, in 200 µL of medium) for 24 h (37 °C, 5% CO_2_). Then, different treatments (PBS, H_2_O_2_ with CFMS‐PVP NSs at pH 7.4 and pH 6.5) were applied to the cells for 12 h. Subsequently the cells were washed several times with PBS and DCFH‐DA solution (0.2 × 10^−6^ m, 1 mL per well) was added to the cells for another 60 min. The presence of intracellular ·OH was examined by the fluorescence of DCFH (*λ*_ex_ = 488 nm, *λ*_em_ = 525 nm).

##### Assessing the Effect of PTT/CDT on Lysosomes and Mitochondria

For the lysosomes damage study, HepG2 cells (1 × 10^5^ cells per dish, 3 mL) were cultured in 35 mm glass‐bottom culture dishes for 24 h. Next, the cells were cultured with DMEM, CFMS‐PVP NSs (30 µg mL^−1^, with the addition of H_2_O_2_ at pH 6.5), CFMS‐PVP NSs (NIR 8 min) for another 24 h. The cells were subsequently incubated with LysoTracker Green DND‐26 (200 µL, 50 × 10^−9^ m) at 37 °C for 15 min in serum‐free DMEM. After washing with PBS three times, the serum‐free DMEM was added to the dishes, and the cells were visualized by CLSM. For the mitochondria damage study, cell culture was performed using the same protocol.

##### Animal Model

Healthy male Balb/c mice (ages 5–6 weeks old) were purchased from Beijing Vital River Laboratory Animal Technology Co., Ltd (Beijing, China). The animal procedures followed the protocols approved by the Animal Care and Use Committee of Peking Union Medical College Hospital. HepG2 cells (1 × 10^7^ cells dissolved in 100 µL PBS) were implanted subcutaneously into the mice to construct the tumor models. The tumor size was measured by a caliper every two days with 16 days’ treatment. Mice with tumor sizes of 70 mm^3^ were selected for experimental studies.

##### PA Imaging

CFMS‐PVP NS suspension (1 mg mL^−1^, 200 µL) were i.v. injected into the tumor‐bearing mice, and PA signals were recorded at 0, 4, 8, 12, and 24 h post‐injection under varied excitation wavelengths (680–900 nm) using a multispectral optical tomography system (MSOT inVision 256, iThera 8 Medical, Germany).

##### In Vivo Toxicity Study

HepG2 tumor‐bearing mice were randomly divided into three groups (6 animals per group): 1) saline (control group), 2) CFMS‐PVP NSs (CDT group), and 3) CFMS‐PVP NSs + 808 nm laser (PTT/CDT group). 200 µL of CFMS‐PVP NS suspension (1 mg kg^−1^) was i.v. injected into tumor‐bearing mice. 8 h post‐injection, for the laser groups the tumor area was irradiated with a 808 nm laser (1.0 W cm^−2^) for 6 min. Real‐time temperature changes and thermal images were recorded using a thermal infrared imaging device. The tumor sizes and mice body weights were recorded every other day and the tumor volume was calculated using the following formula

(9) $$\mathrm{Volume}=\mathrm{Tumor}\;\mathrm{length}\times \frac{\mathrm{Tumor}\;\mathrm{widt}h^{2}}{2}$$

##### Histology

Mice were sacrificed on day 16, and the tumors and major organs were excised and fixed in 4% formalin for histology analysis. The tissues were subject to hematoxylin and eosin (H&E) staining using standard protocols and observed under a digital microscope (Leica DM6000M).

##### ICP Determination

To study the biodistribution of CFMS‐PVP NSs in Balb/c mice, 200 µL of a CFMS‐PVP NS suspension (1 mg mL^−1^) was i.v. injected into tumor‐bearing mice. The mice were sacrificed after 0, 4, 8, 12, and 24 h post‐administration. The organs and excrement were collected, dissected, weighed and digested by nitric acid for distribution studies and ICP (Shimadzu ICPS‐7500) was used to measure the Co concentration.

##### Establishment of a 4T1‐Fluc Tumor Orthotopic Mouse Model

Animals were obtained from Beijing Vital River Laboratory Animal Technology Co., Ltd. (Beijing, China). The animal procedures were carried out in compliance with the protocols approved by Animal Care and Use Committee of Peking Union Medical College Hospital. For orthotopic tumor implantation, a mixture of 4T1 cells (5 × 10^6^, 100 µL in PBS) labeled with Firefly luciferase was injected into the abdomen of mice. Mice with tumor volumes of around 70 mm^3^ were used for experiments.

##### In Vivo Bioluminescence Imaging

4T1‐Fluc tumor‐bearing mice were randomly allocated to three different groups (4 mice per group): 1) saline (control group), 2) CFMS‐PVP NSs (CDT group), and 3) CFMS‐PVP NSs with 808 nm NIR irradiation (PTT/CDT group). 200 µL of the NS suspension at 1 mg mL^−1^ total dose or an equivalent volume of saline were i.v. injected. 8 h post‐injection, the tumor area was irradiated with a 808 nm laser (1.0 W cm^−2^) for 6 min. To monitor tumor growth, the D‐Luciferin probe (150 mg kg^−1^) was intraperitoneally injected into the mice, then mice were anesthetized through isoflurane inhalation (2–3%) for 5 min. Then animals were imaged with the IVIS Lumina fluorescence imaging system every other day.

##### Statistical Analysis

Data are expressed as mean ± standard deviation (S.D). Statistical comparisons were made by unpaired Student's *t*‐test (between two groups) and one‐way ANOVA (for multiple comparisons) followed by Tukey's post‐test: *p < 0.05, **p < 0.01.

##### Data Availability

The data that support the findings of this study are available from the corresponding author on reasonable request.

## Conflict of Interest

The authors declare no conflict of interest.

## Supporting information

Supporting Information
