# Correction to “Multicomponent Transition Metal Dichalcogenide Nanosheets for Imaging‐Guided Photothermal and Chemodynamic Therapy”

**DOI:** 10.1002/advs.202512650

**Published:** 2025-07-17

Adv. Sci. 2020, 7, 2000272.

https://doi.org/10.1002/advs.202000272

In the originally published version of the article, the panels depicting the results from Group 1 in Figure 3E, Group1/Group 3/Group 4 in Figure 3G, and Group 3 in Figure 3H contained some accidental errors. The results and conclusions in the published Article are not changed. The corrected Figure 3 is presented below.


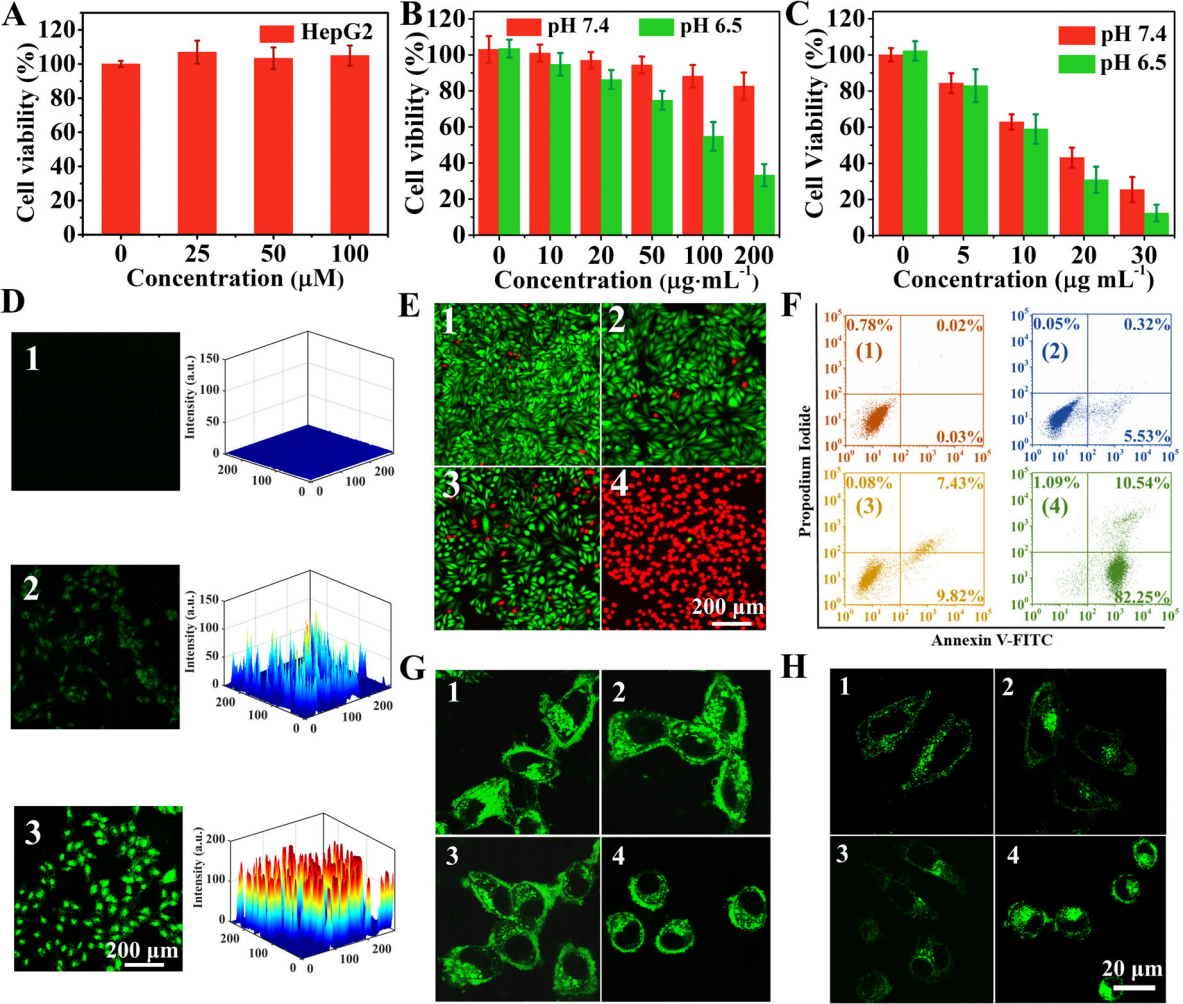


Figure 3. In vitro PTT/CDT studies with HepG2 cells. Relative viabilities of HepG2 cells are shown after incubation A) with different concentrations of H_2_O_2_, and B,C) with Co_2_Fe_0.75_Mn_0.25_S_6_‐PVP NSs and H_2_O_2_ (100 × 10^−6^ M) at different pH values, with B) and without C) 808 nm laser irradiation at 1.0 W cm^−2^ for 8 min. D) Confocal images of DCFH‐DA (2, 7‐dichlorofluorescein diacetate) stained cells. E) Viability of cells in the presence of the CFMS‐PVP NSs. F) Cell apoptosis analysis using the Annexin V‐FITC/PI double staining method: 1) PBS; 2) pH 7.4 + H_2_O_2_ (100 × 10^−6^ M); 3) pH 6.5 + H_2_O_2_ (100 × 10^−6^ M); 4) pH 6.5 + H_2_O_2_ (100 × 10^−6^ M) + NIR (1.0 W cm^−2^, 8 min). Confocal laser scanning microscope (CLSM) images of G) LysoTracker Green DND‐26 (green) stained lysosomes and H) MitoTracker Green FM (green) stained mitochondria. Numbered panels correspond to the following treatments: 1) PBS; 2) pH 7.4 + H_2_O_2_ (100 µM); 3) pH 6.5 + H_2_O_2_ (100 × 10^−6^ M); 4) pH 6.5 + H_2_O_2_ (100 × 10^−6^ µM) + NIR (1.0 W cm^−2^, 8 min).

In addition, there are misuses of the in vivo fluorescence images in Figure 4G for Group 1 (day 0, 2, 8, 12), Group 2 (day 2, 16) and Group 3 (day 8). The results and conclusions in the published Article are not changed. The corrected Figure 4 is shown below.


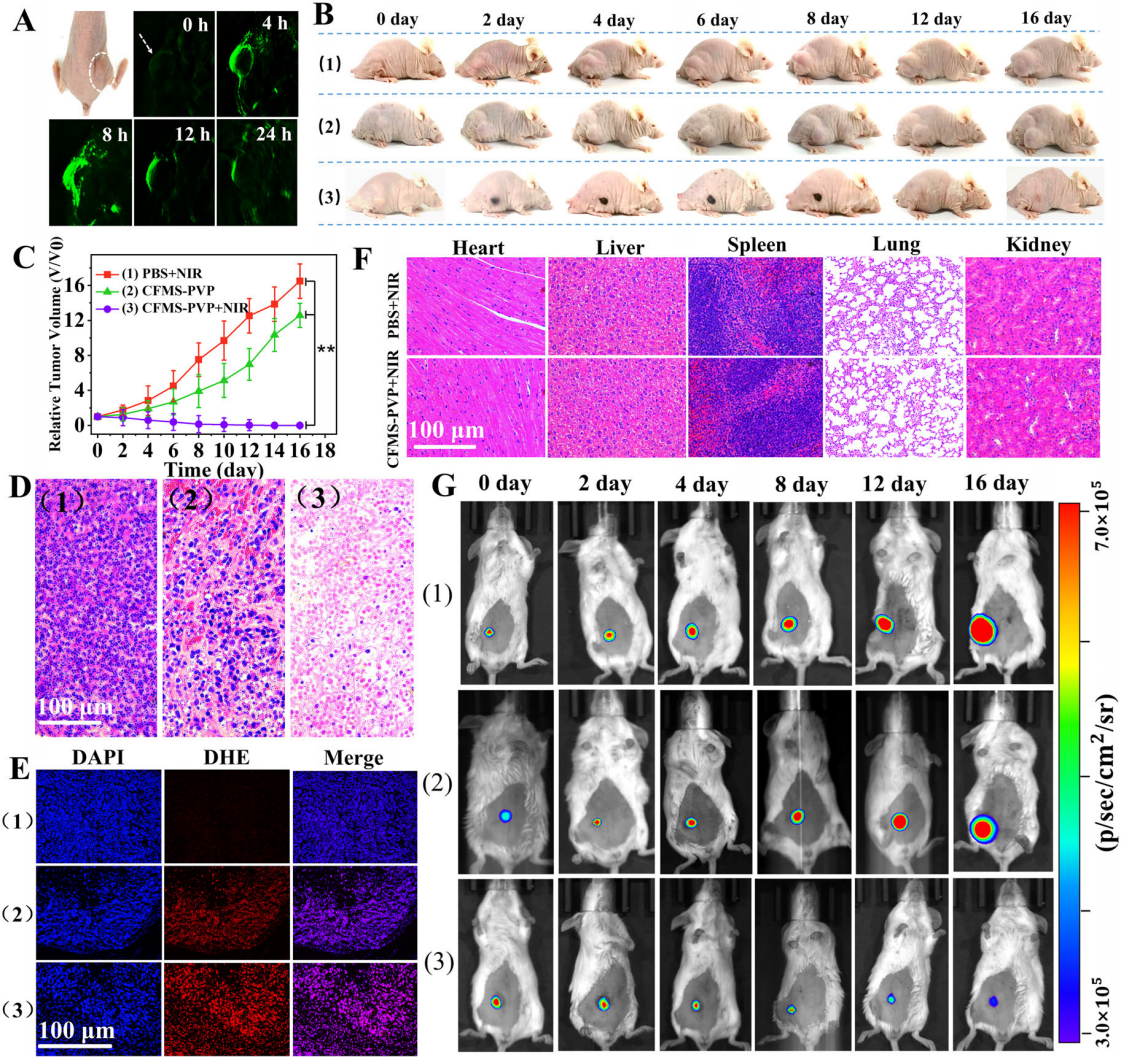


Figure 4. In vivo results in tumor‐bearing mice. Treatment groups are: 1) PBS + NIR, 2) CFMS‐PVP NSs, 3) CFMS‐PVP NSs + NIR. A) Multispectral optical tomography system (MSOT) images of tumor tissue (arrows) at different time points after injection via the tail vein. B) Digital photographs. C) Relative tumor volumes (*n* = 6, mean ± S.D). D) H&E‐stained histological images of the tumor site. E) H&E‐stained histological images of the major organs. F) Fluorescence images of tumor slices after DHE staining (red). The nuclei were stained with DAPI (blue). G) Bioluminescence imaging of 4T1‐Fluc‐tumor‐bearing mice. *p* values were calculated by ANOVA followed by Tukey's post‐test (^*^ *p* < 0.05, ^**^ *p* < 0.01).

We apologize for this error.

