# Supplementary material for: Multicomponent Transition Metal Dichalcogenide Nanosheets for Imaging‐Guided Photothermal and Chemodynamic Therapy
Source: Adv Sci (Weinh). 2020 Sep 30;7(23):2000272. doi: 10.1002/advs.202000272 (PMC7709983; doi:10.1002/advs.202000272)
Supplement: Supplementary file 1 — Supporting Information [file ADVS-7-2000272-s001.pdf]

WILEY-VCH

Copyright WILEY-VCH Verlag GmbH & Co. KGaA, 69469 Weinheim, Germany, 2018.

Supporting Information

Multicomponent Transition Metal Dichalcogenide Nanosheets for Imaging-Guided Photothermal and Chemodynamic Therapy

Yu Zhu,‡ Yingjie Wang,‡ Gareth R. Williams, Liyang Fu, Jingjing Wu, Hui Wang, Ruizheng Liang, Xisheng Weng,* Min Wei**

Y. Zhu, L. Fu, J. Wu, H. Wang, Dr. R. Liang, Prof. M. Wei
State Key Laboratory of Chemical Resource Engineering, Beijing Advanced Innovation Center for Soft Matter Science and Engineering, Beijing University of Chemical Technology, Beijing 100029, P. R. China
E-mail: liangrz@mail.buct.edu.cn (Dr. R. Liang); weimin@mail.buct.edu.cn (Prof. M. Wei)

Y. Wang, Prof. X. Weng
Department of Orthopaedics, Peking Union Medical College Hospital, Peking Union Medical College & Chinese Academy of Medical Sciences, Beijing 100730, China
E-mail: xshweng@medmail.com.cn (Prof. X. Weng)

Dr. G. R. Williams
UCL School of Pharmacy, University College London, 29-39 Brunswick Square, London WC1N 1AX, UK

‡ These authors contributed equally to this work.

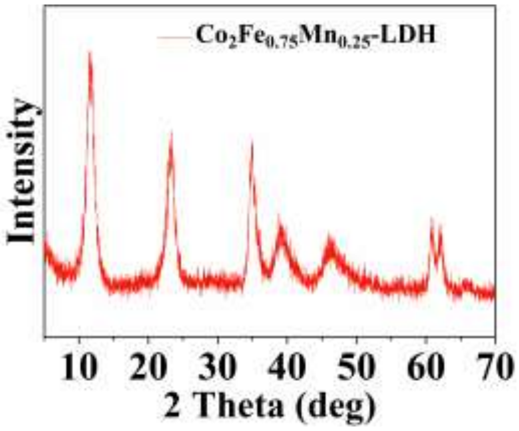

Figure S1. XRD pattern of CoFeMn-LDH precursor with Co: Fe: Mn = 2: 0.75: 0.25.

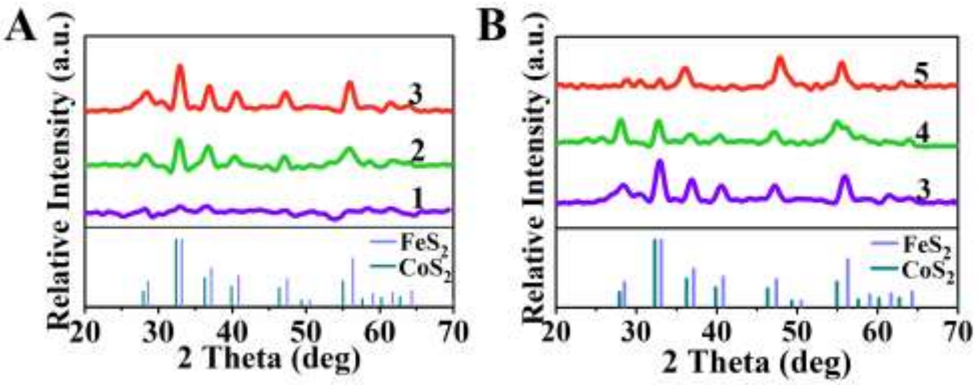

Figure S2. XRD patterns of CFMS NSs with Co: Fe: Mn = 1: 0.25: 0.75 (line 1), 1: 0.5: 0.5 (line 2), 1: 0.75: 0.25 (line 3), 2: 0.75: 0.25 (line 4), and 3: 0.75: 0.25 (line 5).

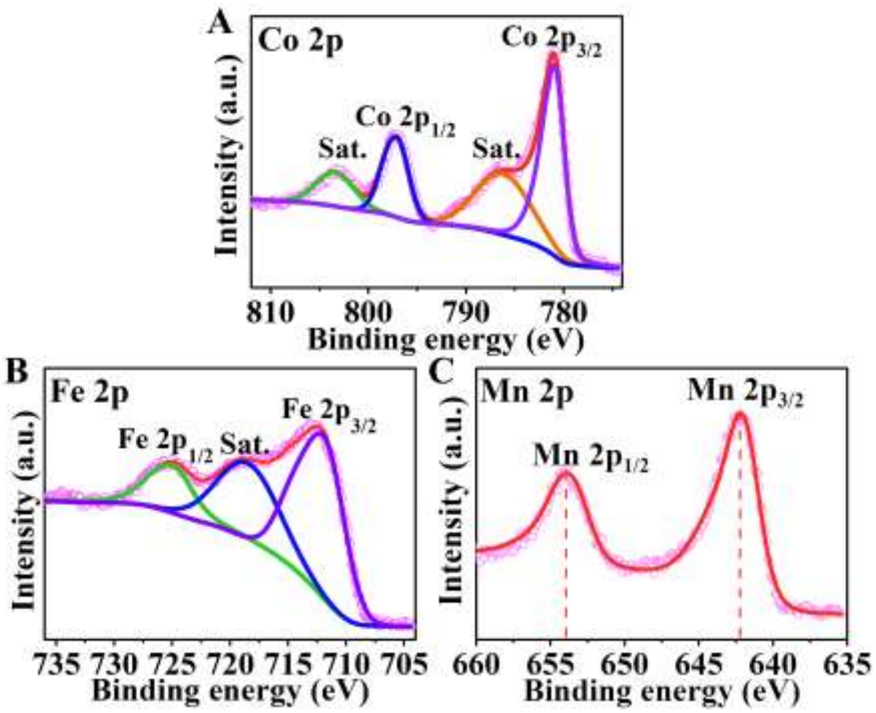

Figure S3. XPS spectra of the A) Co 2*p*, B) Fe 2*p*, C) Mn 2*p* regions for $Co_2Fe_{0.75}Mn_{0.25}$-LDH.

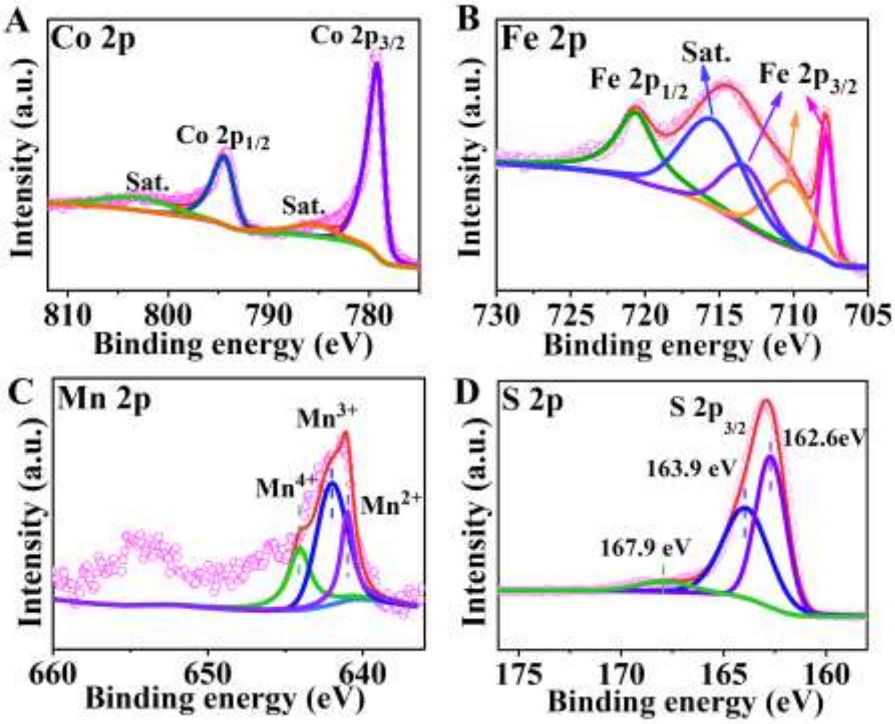

Figure S4. XPS spectra of the A) Co 2*p*, B) Fe 2*p*, C) Mn 2*p*, D) S 2*p* regions for CFMS NSs (Co: Fe: Mn = 2: 0.75: 0.25).

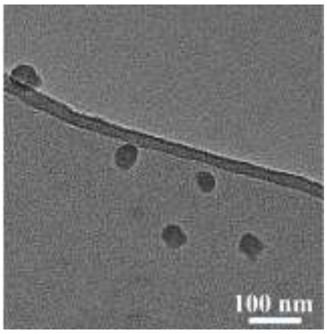

Figure S5. TEM image of CFMS-PVP NSs (Co: Fe: Mn = 2: 0.75: 0.25).

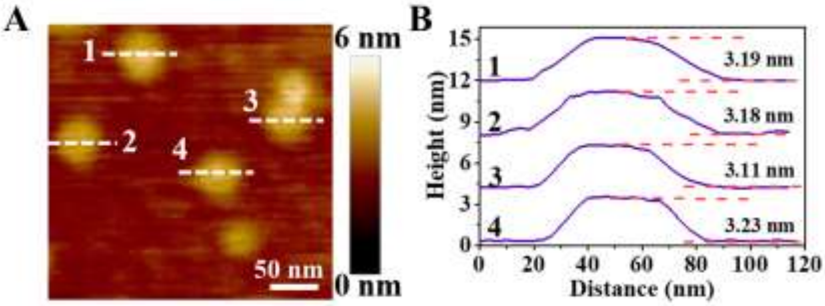

Figure S6. A) AFM image and B) measured thickness of CFMS-PVP NSs (Co: Fe: Mn = 2: 0.75: 0.25).

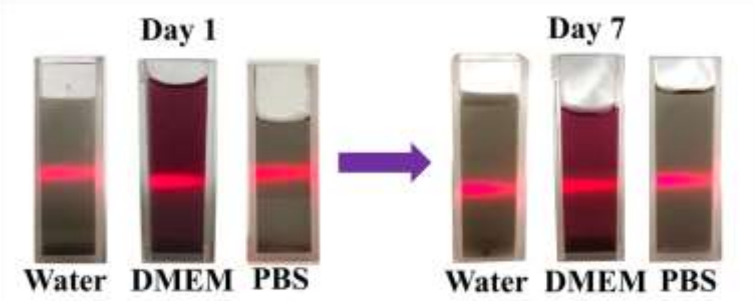

Figure S7. The Tyndall effect of $Co_2Fe_{0.75}Mn_{0.25}S_6$-PVP NSs in water, DMEM and PBS from Day 1 to Day 7.

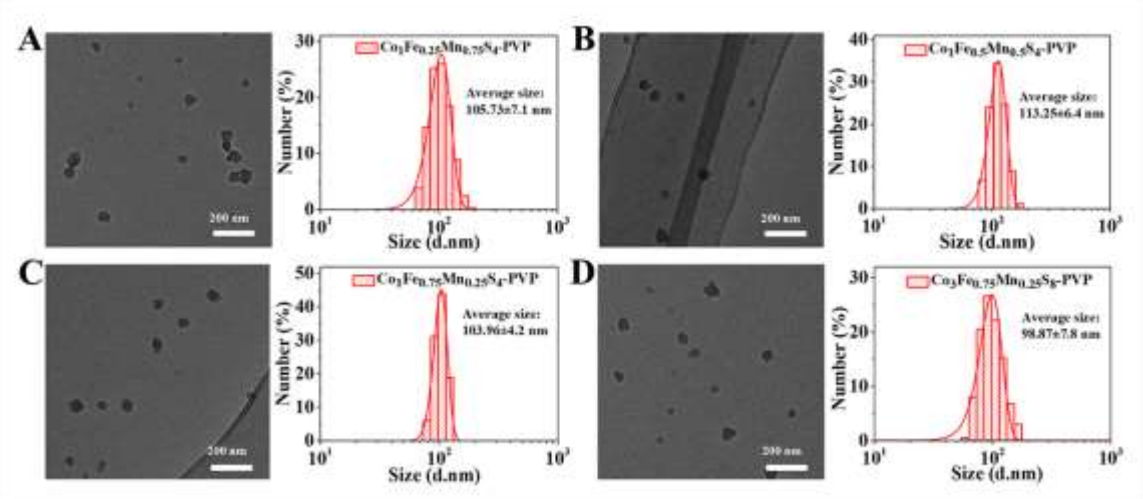

Figure S8. TEM image and corresponding size distribution of CFMS-PVP NSs with various ratios A) Co: Fe: Mn = 1: 0.25: 0.75, B) Co: Fe: Mn = 1: 0.5: 0.5, C) Co: Fe: Mn = 1: 0.75: 0.25, D) Co: Fe: Mn = 3: 0.75: 0.25.

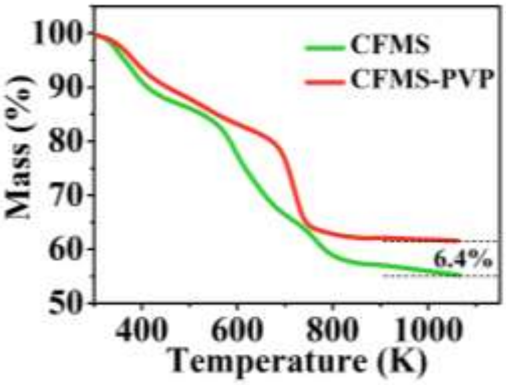

Figure S9. Thermogravimetric curves of CFMS and CFMS-PVP NSs (Co: Fe: Mn = 2: 0.75: 0.25).

Table S1. Chemical composition of CFMS-PVP NSs with various molar ratios of Co/Fe/Mn and S/(Co + Fe + Mn), based on ICP results.

CFMS-PVP	Co/Fe/Mn	S/Metal
1/0.25/0.75/4	1/0.28/0.74	2.11
1/0.5/0.5/4	1/0.61/0.57	2.05
1/0.75/0.25/4	1/0.68/0.24	2.07
2/0.75/0.25/6	2/0.76/0.28	2.31
3/0.75/0.25/8	3/0.81/0.27	2.21

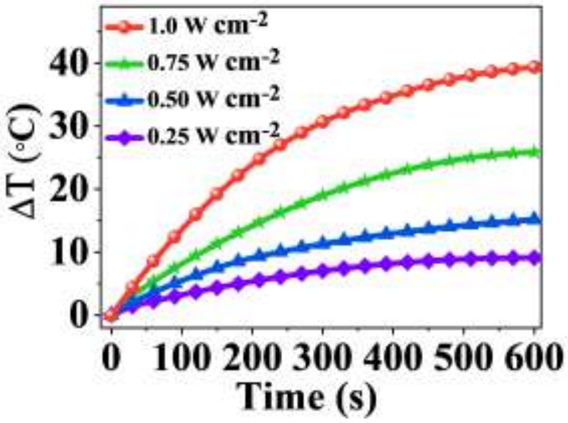

Figure S10. Temperature profiles of $Co_2Fe_{0.75}Mn_{0.25}S_6$-PVP NSs dispersion (50 μg mL^{-1}) under 808 nm laser irradiation at various power densities (0.25, 0.50, 0.75, and 1.0 W cm^{-2}).

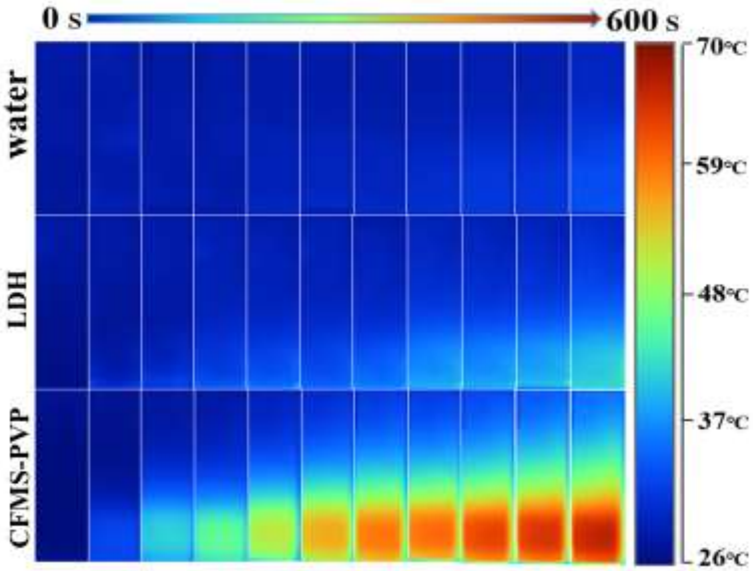

Figure S11. The photothermal photographs of water, $Co_2Fe_{0.75}Mn_{0.25}$-LDH precursor, CFMS-PVP NSs (Co: Fe: Mn = 2: 0.75: 0.25) detected by a thermal infrared imaging device.

Table S2. Photothermal conversion efficiency (η) of different PTT agents reported in the literature.

Photothermal agent	η (%)	Size	*Refs*
MoS_2-PEG NSs	32.96%	~80 nm	*ACS Nano*. 13, 2544−2557 (2019)
WS_2-PVP NSs	36.9%	~200 nm	*Adv. Funct. Mater.* 29, 1901722 (2019)
TaS_2-PEG NSs	39.0%	110 nm	*Adv. Funct. Mater.* 27, 1703261 (2017)
$Ti_{0.71}Ta_{0.29}S_yO_z$ NSs	39.2%	0.2~2 μm	*Angew. Chem. Int. Ed.* 56, 7842 – 7846 (2017)
Cu_2Se	50.89%	~86.89 nm	*Chem. Mater.* 31, 6174−6186 (2019)
Cu-Fe-Se NSs	78.9%	70 nm	*ACS Appl. Mater. Interfaces.* 10, 43396−43404 (2018)
ReS_2 NSs	79.2%	~100 nm	*Small.* 14, 1703789 (2018)
CFMS-PVP NSs	89.0%	~60.0 nm	This work

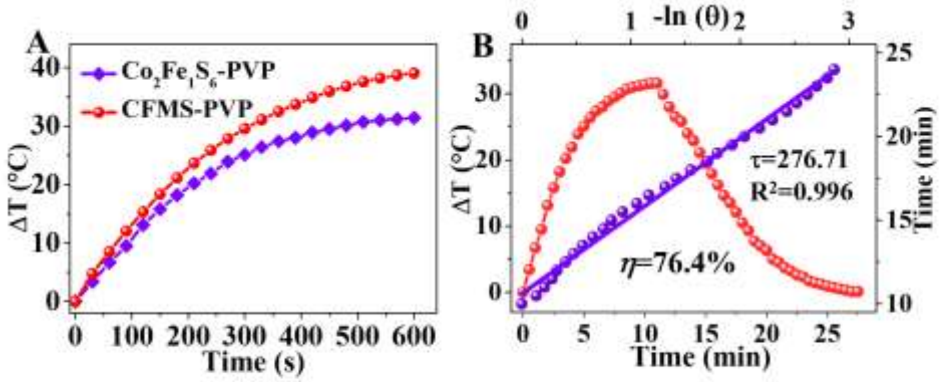

Figure S12. A) Photothermal heating curves of $Co_2Fe_{0.75}Mn_{0.25}S_6$-PVP NSs and $Co_2Fe_1S_6$-PVP NSs. B) Calculation of the photothermal conversion efficiency for $Co_2Fe_1S_6$-PVP NSs at 808 nm. The time constant (τ_s) for the heat transfer was calculated from the cooling period (purple line).

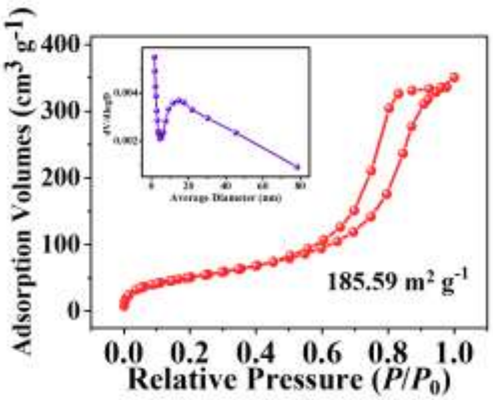

Figure S13. N_2 adsorption-desorption isotherm of the $Co_2Fe_{0.75}Mn_{0.25}S_6$-PVP NSs. Inset: corresponding pore size distribution.

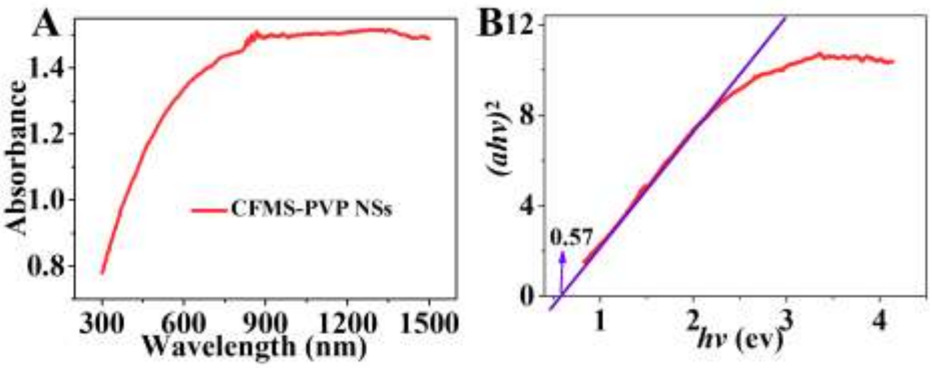

Figure S14. A) Diffuse reflection spectrum of the solid $Co_2Fe_{0.75}Mn_{0.25}S_6$-PVP NSs. B) The responding Tauc plot of $(\alpha hv)^2$ *vs*. photons energy (*hv*).

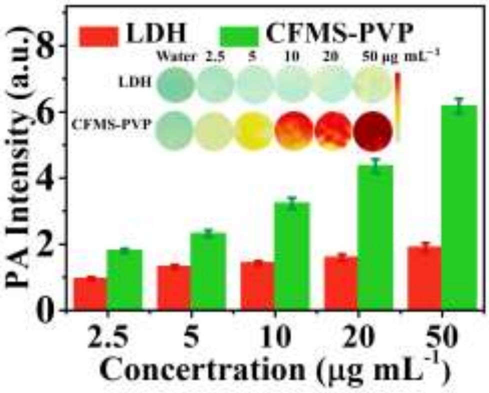

Figure S15. PA intensity of $Co_2Fe_{0.75}Mn_{0.25}S_6$-PVP NSs and $Co_2Fe_{0.75}Mn_{0.25}$-LDH aqueous suspensions at various concentrations (2.5, 5, 10, 20, and 50 μg mL^{-1} from left to right). Data are given as mean ± S.D. (n = 3).

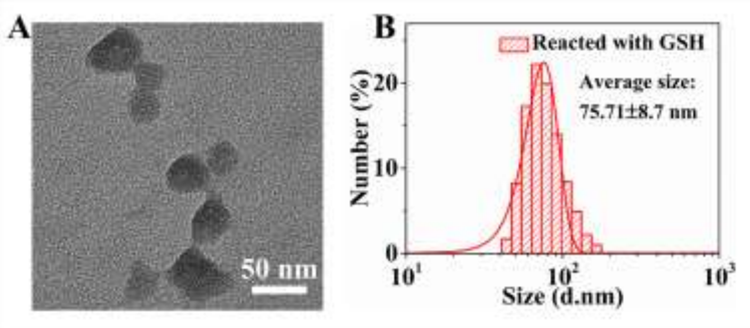

Figure S16. TEM image and corresponding size distribution of $Co_2Fe_{0.75}Mn_{0.25}S_6$-PVP NSs (50 μg mL^{-1}) reacted with GSH (1.0 mM).

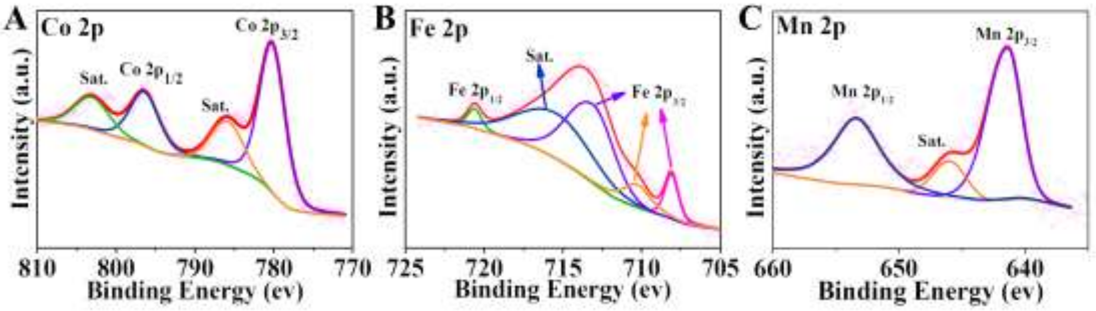

Figure S17. A) Co 2*p*, B) Fe 2*p*, C) Mn 2*p* XPS spectra for CFMS-PVP NSs (Co: Fe: Mn = 2: 0.75: 0.25) after GSH treatment.

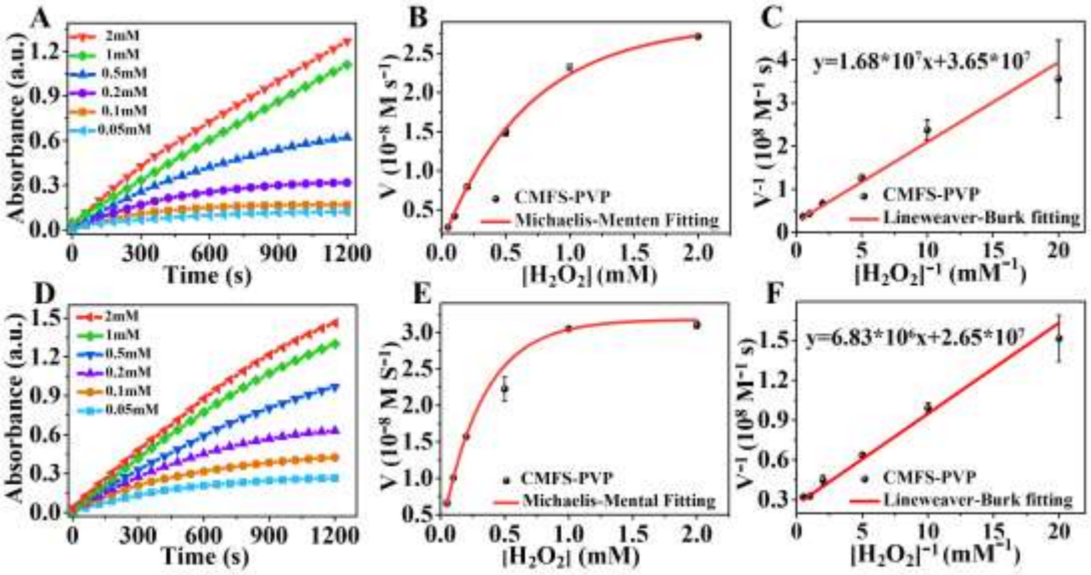

Figure S18. Fenton reactions occurring in the presence of $Co_2Fe_{0.75}Mn_{0.25}S_6$-PVP NSs. Michaelis-Menten steady-state kinetics upon the addition of varied concentration of H_2O_2 (0.05, 0.1, 0.2, 0.5, 1.0 and 2.0 mM) are shown at 298 K (A-C) and 318 K (D-F). Data are plotted as mean ± S.D. (n = 3).

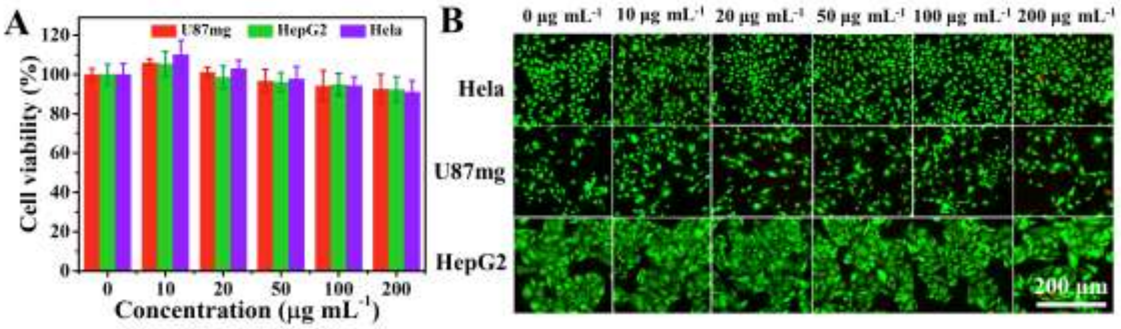

Figure S19. Cell viability of Hela, U87mg, and HepG2 cells incubated with various concentrations of $Co_2Fe_{0.75}Mn_{0.25}S_6$-PVP NSs. (Data are given as mean ± S.D, $n = 6$).

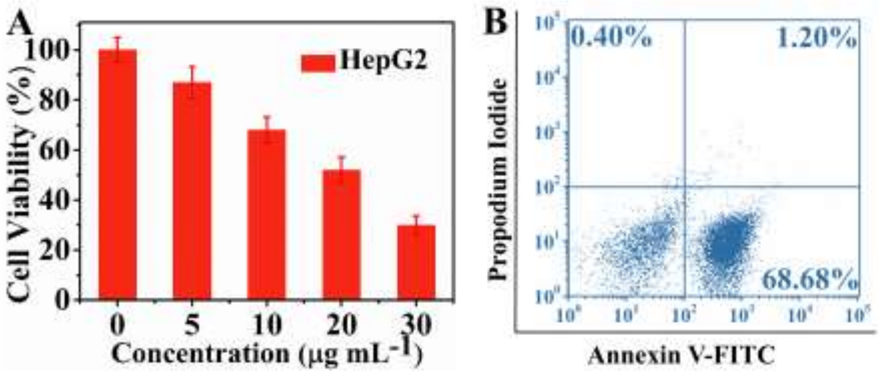

Figure S20. A) Relative viabilities of HepG2 cells after incubation with different concentrations of $Co_2Fe_{0.75}Mn_{0.25}S_6$-PVP NSs and 808 nm laser irradiation at 1.0 W cm^{-2} for 8 min. B) Cell apoptosis analysis using the Annexin V-FITC/PI double staining method with 808 nm laser irradiation at 1.0 W cm^{-2} for 8 min.

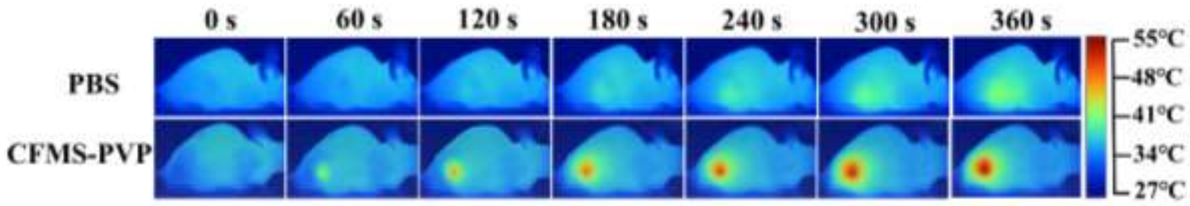

Figure S21. Thermal infrared imaging at the tumor site of HepG2-tumor bearing mice exposed to 808 nm laser irradiation for 6 min at 8 h post injection.

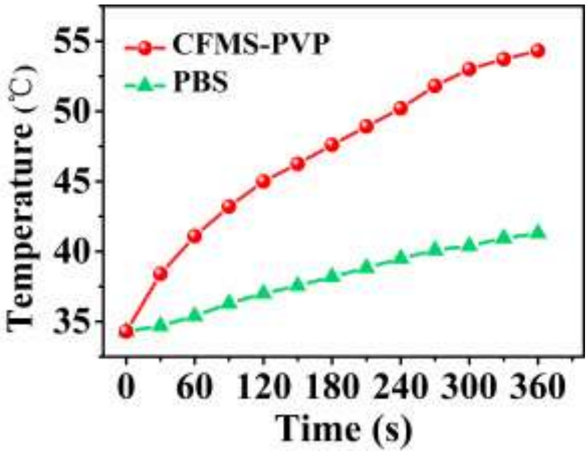

Figure S22. Temperature rise on tumors of mice administered by *i.v.* injection with PBS, and $Co_2Fe_{0.75}Mn_{0.25}S_6$-PVP NSs under 808 nm laser irradiation at 1.0 W cm^{-2} for 6 min.

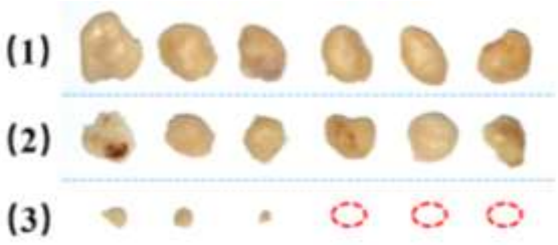

Figure S23. Digital photographs of the excised tumors from each group after 16 days of therapy. Groups: (1) PBS + NIR (6 min irradiation, control group), (2) CFMS-PVP NSs (CDT alone), (3) CFMS-PVP NSs + NIR (6 min irradiation, PTT/CDT).

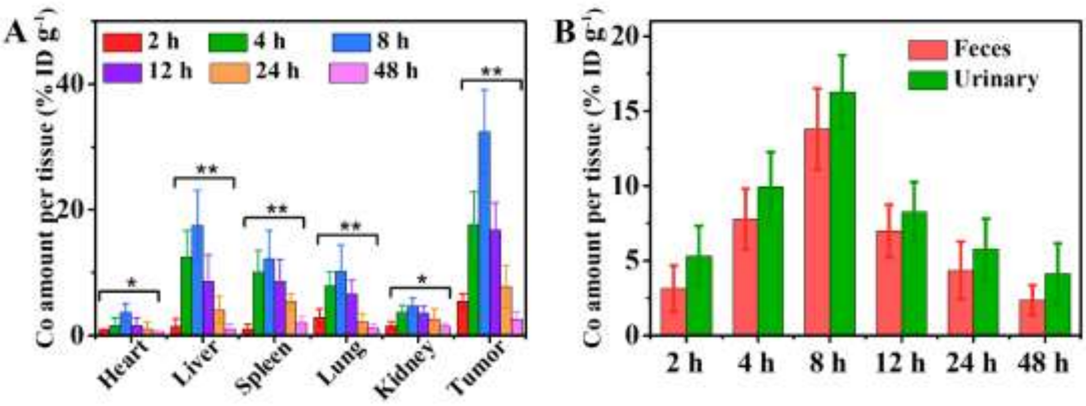

Figure S24. A) The distribution of the $Co_2Fe_{0.75}Mn_{0.25}S_6$-PVP NSs in various organs as a function of time, determined by measuring Co concentrations. B) Excretion of the CFMS-PVP NSs, again quantified *via* Co concentration. Data are presented as mean ± S.D, n = 3. P values in (A) were calculated by ANOVA followed by Tukey's post-test (*P < 0.05, **P < 0.01).

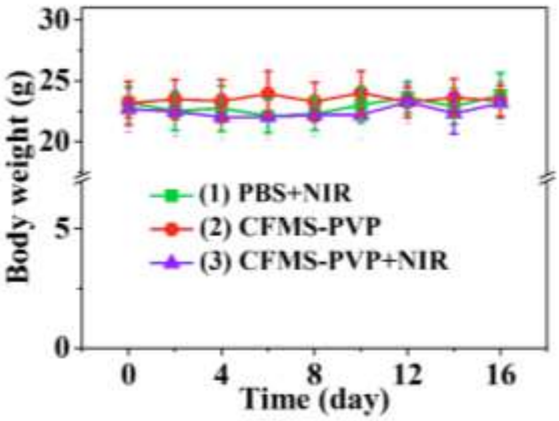

Figure S25. Body weight changes of HepG2 tumor bearing mice as a function of time after various treatments. Data are presented as mean ± S.D, n = 6.

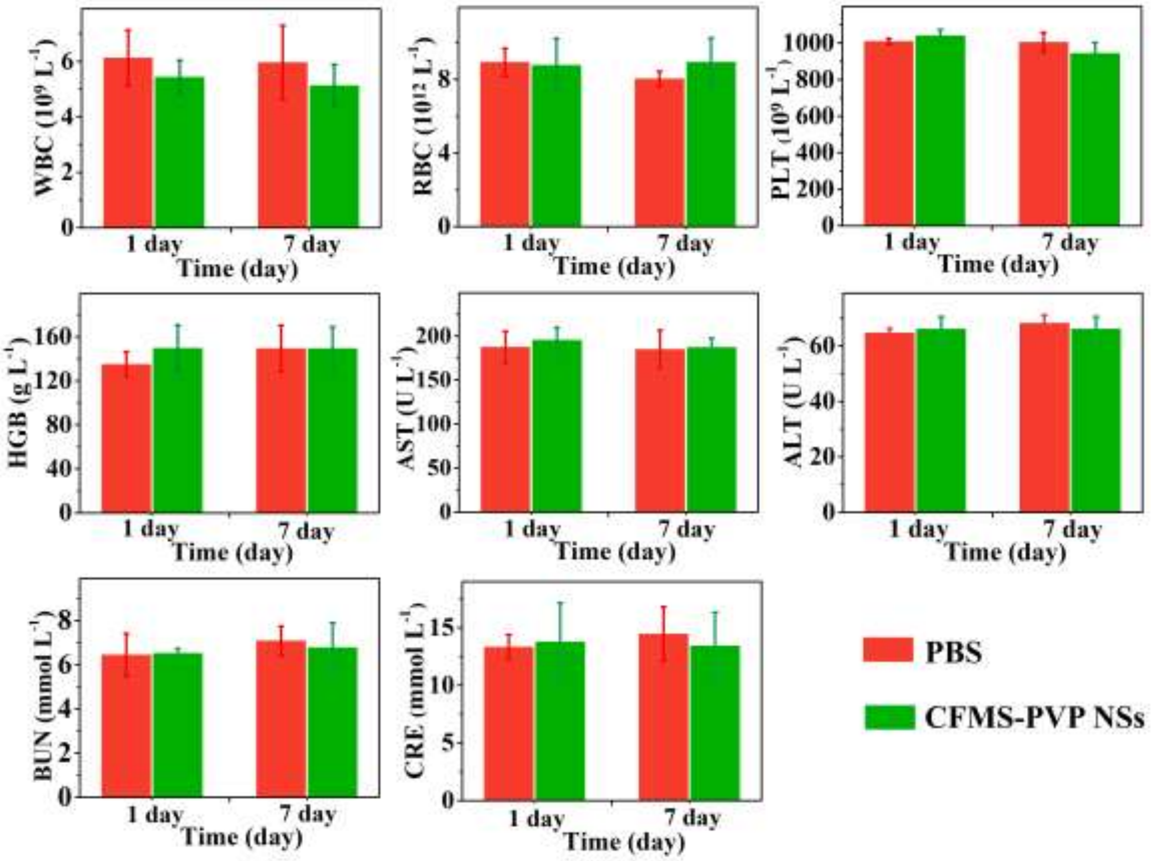

Figure S26. Kidney and liver function markers and blood cell counts of nude mice bearing HepG2 tumors after injection of PBS (control) and CFMS-PVP NSs. Data are presented as mean ± S.D, n = 3.

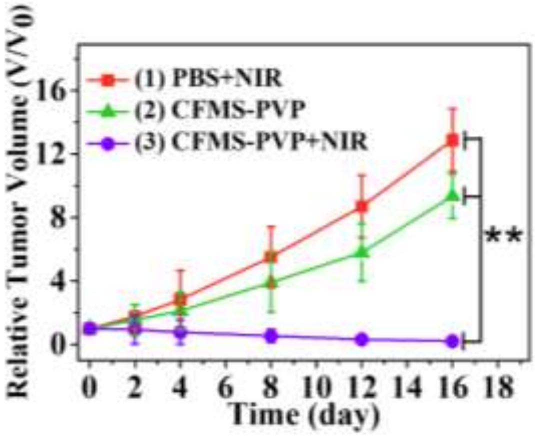

Figure S27. Relative tumor volume of 4T1-Fluc-tumor-bearing mice after different treatments. Data are presented as mean ± S.D, n = 4. P values were calculated by ANVOA followed by Tukey's post-test (*P < 0.05, **P < 0.01).
